# The impact of iterative removal of low-information cluster-period cells from a stepped wedge design

**DOI:** 10.1186/s12874-023-01969-7

**Published:** 2023-07-06

**Authors:** Ehsan Rezaei-Darzi, Kelsey L. Grantham, Andrew B. Forbes, Jessica Kasza

**Affiliations:** School of Public Health and Preventive Medicine, Monash University, Melbourne, Australia

**Keywords:** Longitudinal cluster randomised trials, Correlation structure, Highly efficient design, Incomplete design, Discrete-time decay

## Abstract

**Background:**

Standard stepped wedge trials, where clusters switch from the control to the intervention condition in a staggered manner, can be costly and burdensome. Recent work has shown that the amount of information contributed by each cluster in each period differs, with some cluster-periods contributing a relatively small amount of information. We investigate the patterns of the information content of cluster-period cells upon iterative removal of low-information cells, assuming a model for continuous outcomes with constant cluster-period size, categorical time period effects, and exchangeable and discrete-time decay intracluster correlation structures.

**Methods:**

We sequentially remove pairs of “centrosymmetric” cluster-period cells from an initially complete stepped wedge design which contribute the least amount of information to the estimation of the treatment effect. At each iteration, we update the information content of the remaining cells, determine the pair of cells with the lowest information content, and repeat this process until the treatment effect cannot be estimated.

**Results:**

We demonstrate that as more cells are removed, more information is concentrated in the cells near the time of the treatment switch, and in “hot-spots” in the corners of the design. For the exchangeable correlation structure, removing the cells from these hot-spots leads to a marked reduction in study precision and power, however the impact of this is lessened for the discrete-time decay structure.

**Conclusions:**

Removing cluster-period cells distant from the time of the treatment switch may not lead to large reductions in precision or power, implying that certain incomplete designs may be almost as powerful as complete designs.

**Supplementary Information:**

The online version contains supplementary material available at 10.1186/s12874-023-01969-7.

## Background

**Fig. 1:**
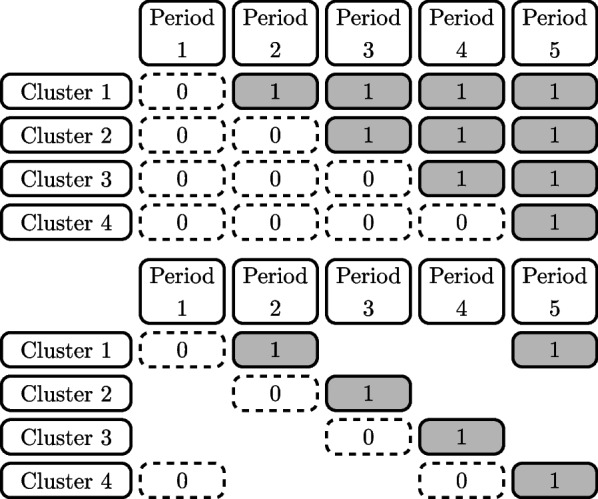
Top: A complete standard stepped wedge design schematic with four clusters (rows) and five periods (columns). Bottom: An incomplete stepped wedge design schematic with four clusters and spanning five periods. Cluster-period cells contain either 0, indicating the control condition, or 1, indicating the intervention condition. Blank areas within the schematic indicate cluster-periods in which no measurements are taken

Stepped wedge designs are a particular type of longitudinal cluster randomised trial design that are being increasingly used to evaluate interventions in public health and related fields [1]. All clusters commence in the control condition and cross over to the intervention condition in a staggered manner until all clusters implement the intervention condition by the final period. An example of a schematic of a stepped wedge design is provided in the top panel of Fig. 1. The requirement that clusters measure participants’ outcomes in each period can make these trials particularly burdensome and expensive to clusters, and, when a cohort sampling structure is employed, to participants as well [2]. However, stepped wedge designs have several beneficial characteristics: they are useful for testing interventions that cannot be undone or that must be gradually rolled out across a system, and they ensure that all clusters receive the intervention during the course of the trial.

Recent work has shown that the cluster-period cells in the stepped wedge design differ in the amount of information they contribute to estimation of the treatment effect [3]. The “information content” of a cluster-period cell was defined as the ratio of the variance of the treatment effect estimator for the resulting design when that cell is removed to the variance of the treatment effect estimator for the complete design [3–5]. A key finding from these papers is that some cluster-periods contribute a relatively small amount of information about the treatment effect. This strongly suggests that “incomplete” designs where not all clusters contribute measurements in all periods (e.g., bottom panel of Fig. 1), may still provide sufficient power to detect effects of interest. The observed patterns of information content of *individual* cells have previously been used to provide a basis for the selection of incomplete stepped wedge designs; in Kasza et al. [5], four incomplete designs where clusters would provide measurements in a limited number of pre- and post-treatment switch periods were considered. However, how the information content changes when sets of two or more cells are removed from the design has not been considered. Further investigation of the properties of the information content of cluster-period cells of stepped wedge designs is needed to guide the selection of incomplete stepped wedge designs; such incomplete designs may be more feasible for trialists than a complete stepped wedge.

Although incomplete stepped wedge designs have been considered in the statistical literature, e.g. Hemming et al. [6] and Unni et al. [7], like Kasza et al. [5], these have focused on a limited selection of prespecified designs and were not based on any information content considerations. For example, Hemming et al. [6] and Unni et al. [7] considered a design with one control period followed by two intervention periods in each sequence and stepped wedge designs with a transition or learning period in which no measurements are taken between the control and intervention periods. A more general investigation of a range of incomplete stepped wedge designs was conducted by Hooper et al. [8] for trials with a continuous outcome and continuous recruitment of participants. The authors sought to identify incomplete designs obtained by removing participants yielding the lowest reduction in the precision of the treatment effect estimator. They developed an algorithm to minimise the number of participants in a design by removing two *participants* from a complete design at a time (where the two participants were selected so that the resulting design maintained a “skew-symmetric” structure [9]).

In this paper we will use the information content metric to guide the exploration of a range of progressively reduced stepped wedge designs, starting with a complete design and removing cluster-period cells until a minimally viable incomplete design is obtained (i.e. the smallest design that can provide an estimate of the treatment effect). We progress through incomplete designs by removing cluster-period cells with low information content. We then assess the patterns of information content across these designs, how much precision is lost as we remove cells, and how study power correspondingly reduces. We have two aims in this work. First, we wish to obtain a better understanding of how the pattern of information content of cluster-period cells of a stepped wedge design changes as cells with low information content are removed. Second, we provide an approach to help researchers identify less burdensome designs that maintain power similar to complete stepped wedge designs.

Our paper is organised as follows: in the Methods Section, we describe our statistical model for continuous outcomes, intracluster correlation structures, and provide a general expression for the variance of the treatment effect estimator valid for complete and incomplete designs. We also provide a more general definition of information content than has been considered previously, outline our procedure for removing low information content cells, and define a metric for comparing the precision of designs. In the Results Section, we implement our procedure for some specific trial design examples and describe the information content patterns and corresponding changes in design precision and power. We also assess the impact on design precision of removing prespecified proportions of cluster-period cells across a range of trial configuration parameters. We conclude with a discussion of our findings in the last section.

## Methods

### A statistical model for continuous outcomes

We consider stepped wedge designs with different sets of participants in each period, often termed repeated cross-sectional sampling. We define $Y_{kji}$ as the continuous measured outcome for participant $i=1,\dots ,m$ at time $j=1,\dots ,T$ in cluster $k=1,\dots ,K$. The model that we consider applies to all longitudinal cluster randomised designs. For simplicity we assume that one cluster is randomised to each sequence so that $K=T-1$. We consider two intracluster correlation structures: the exchangeable structure and the discrete-time decay correlation structure. The exchangeable correlation structure assumes that the correlation between the outcomes of any pair of participants in the same cluster is identical regardless of the distance in time between their measurements. This was first implemented via a linear mixed model in a seminal paper on analysis of stepped wedge designs [10]. The discrete-time decay correlation structure allows the correlation between participants’ outcomes to depend on their periods of measurement, with decreasing correlation as the time between their periods of measurement increases [4]. Underlying linear mixed effects models yielding these two correlation structures can be represented as:

1 $$\begin{aligned} Y_{kji}= & {} \mu + \beta _{j} + X_{kj}\theta + \alpha _{k} + \epsilon _{kji}, \nonumber \\ \quad{} & {} \alpha _{k} \sim N\left( 0,\tau ^{2}\right) , \quad \epsilon _{kji} \sim N\left( 0, \sigma ^{2}_{\epsilon }\right) \end{aligned}$$

2 $$\begin{aligned} Y_{kji}= & {} \mu + \beta _{j} + X_{kj}\theta + \gamma _{kj} + \epsilon _{kji}, \nonumber \\ \quad{} & {} \mathbf {\gamma }_{k} \sim N_{T}\left( 0,\tau ^{2}\textbf{R}\right) , \quad \epsilon _{kji} \sim N\left( 0, \sigma ^{2}_{\epsilon }\right) \end{aligned}$$

where $\beta _{j}$ is the fixed time effect for period *j*, with $\beta _{1}=0$ for identifiability; $\mu$ is the overall mean outcome in period 1; $X_{kj}$ is the intervention indicator variable, equal to 0 when cluster *k* at period *j* is in the control condition, and 1 when cluster *k* at period *j* is in the treatment condition; $\theta$ is the treatment/intervention effect of interest, $\alpha _{k}$ is the random effect for cluster *k* for the exchangeable model (1), and $\mathbf {\gamma }_{k}=\left( \gamma _{k1},\dots ,\gamma _{kT} \right) ^{T}$ is the vector of cluster-period random effects for the discrete-time decay model (2) with covariance matrix $\tau ^2 \textbf{R}$ (elements of $\textbf{R}$ described below), and $\epsilon _{kji}$ is the subject-specific random error. In model (2), the correlation between different subjects’ outcomes measured in periods *j* and *s* within a cluster is assumed to depend on the time between these periods: $cov\left( \gamma _{kj},\gamma _{ks}\right) =\tau ^2 r^{\left| j-s\right| }$ and so $corr\left( Y_{kji},Y_{ksl}\right) =\frac{\tau ^2}{\tau ^2+\sigma ^{2}_{\epsilon }} r^{\left| j-s\right| }$ = $\rho r^{\left| j-s\right| }$, $0 < r \le 1$. That is, the $\left( j,s\right)$ element of $\textbf{R}$ is given by $r^{\left| j-s\right| }$. We refer to $\rho$ as the within-period intracluster correlation (ICC), representing the correlation between two subjects’ outcomes measured within the same cluster in the same period. We refer to the parameter *r* as the cluster autocorrelation (CAC), representing the proportionate reduction in correlation from one period to the next. Note that model (1) is a special case of model (2), and is returned when $r=1$: $corr(Y_{kji},Y_{kjl})=corr\left( Y_{kji},Y_{ksl}\right) =\rho .$

#### Variance of the treatment effect estimator for incomplete designs

When the correlation structure and correlations are known (as is assumed when calculating study power), a common approach for estimating the treatment effect $\theta$ uses an estimator $\hat{\theta }$ obtained via generalised least squares. The variance of the treatment effect estimator, $var(\hat{\theta })$, is a key ingredient in calculating the required sample size and power of the trial and we therefore focus on this quantity at the trial design stage.

Letting $\bar{Y}_{kj}=\frac{1}{m} \sum _{i=1}^{m}Y_{kji}$ be the mean outcome in cluster *k* in period *j* and $\bar{\textbf{Y}}_{k}=\left( \bar{Y}_{k1},\dots ,\bar{Y}_{kT} \right) ^{T}$ be the vector of cluster-period means for cluster *k*, then the covariance matrix for a cluster, assumed common across clusters, is given by ${\textbf {V}}_{\bar{Y}}=\frac{\sigma ^{2}_{\epsilon }}{m}\textbf{I}_{T \times T}+\tau ^{2} \textbf{R}$ where $\textbf{I}_{T \times T}$ is the $T\times T$ identity matrix. When $r=1$, the matrix $\textbf{R}=\textbf{J}_{T \times T}$, a $T\times T$ matrix of ones, and this reduces to model (1). Letting ${\textbf {X}}_{k}=\left( X_{k1},\dots ,X_{kT}\right) ^{T}$ be the vector of treatment indicators for cluster *k*, the variance of the treatment effect estimator can then be represented as [3]:

3 $$\begin{aligned} var\hat{(\theta )}= & {} \left\{ \sum \limits _{k=1}^{K}{\textbf {X}}^T_{k} {\textbf {V}}^{-1}_{\bar{Y}} {\textbf {X}}_{k} - \frac{1}{K} \left( \sum \limits _{k=1}^{K} X_{k1},\dots , \sum \limits _{k=1}^{K} X_{kT}\right) \right. \nonumber \\{} & {} \left. \times {\textbf {V}}_{\bar{Y}}^{-1} \left( \sum \limits _{k=1}^{K} X_{k1},\dots , \sum \limits _{k=1}^{K} X_{kT}\right) ^{T}\right\} ^{-1}. \end{aligned}$$

This expression is valid for complete stepped wedge designs but a more general expression is required for incomplete designs. Letting $T_{k}$ represent the number of measurement periods in the sequence assigned to cluster *k*, ${\textbf {Z}}_{k}$ be the $T_{k} \times T$-dimensional matrix encoding the parameterisation of the time effects corresponding to cluster *k*, resembling a $T \times T$ identity matrix with rows corresponding to unobserved periods deleted, ${\textbf {V}}_{k}$ be the $T_{k} \times T_{k}$ covariance matrix for cluster *k* and ${\textbf {X}}_{k}$ be the $T_{k} \times 1$-dimensional column vector of treatment indicators for the measurement periods for cluster *k*, then a more general expression for the variance of the treatment effect estimator is given by:

4 $$\begin{aligned} var\hat{(\theta )}= & {} \left\{ \sum \limits _{k=1}^{K}{\textbf {X}}^T_{k} {\textbf {V}}^{-1}_{k} {\textbf {X}}_{k} - \left( \sum \limits _{k=1}^{K} {\textbf {Z}}^T_{k}{\textbf {V}}^{-1}_{k} {\textbf {X}}_{k}\right) ^T \right. \nonumber \\{} & {} \left. \times \left( \sum \limits _{k=1}^{K} {\textbf {Z}}^T_{k} {\textbf {V}}^{-1}_{k} {\textbf {Z}}_{k} \right) ^{-1} \left( \sum \limits _{k=1}^{K}{\textbf {Z}}^T_{k} {\textbf {V}}^{-1}_{k} {\textbf {X}}_{k} \right) \right\} ^{-1}. \end{aligned}$$

This expression is valid for both complete and incomplete stepped wedge designs. Note that the standard variance expression for complete designs, equation (3), is returned if all cluster-periods are measured, which would mean that the ${\textbf {Z}}_{k}$ matrices are all identity matrices, and all clusters would have the same covariance matrix for a cluster, ${\textbf {V}}_{\bar{Y}}$. The derivation for equation (4) is provided in Section A of Additional file 1.

### Obtaining and evaluating progressively reduced designs

#### Information content of pairs of cells

**Fig. 2:**
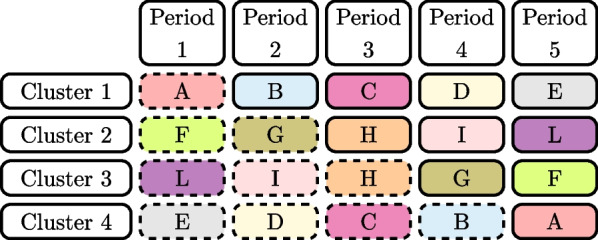
Centrosymmetric cells for the standard stepped wedge design with four clusters and five periods. Letters and colours represent pairs of centrosymmetric cells

The information content metric that was introduced by Kasza et al. [3] was used to identify the amount of information that each individual cell, period or cluster of a stepped wedge design contributes to estimation of the treatment effect. It was defined as the ratio of the variance of the treatment effect estimator for the design when a cell is removed to that of the variance of the treatment effect estimator for the complete design. However, since Kasza et al. [3] also proved that the information content is equal for cells in a centrosymmetric pair for the intracluster correlation structures we assume, we will instead consider the information content of centrosymmetric *pairs* of cells. Informally, a centrosymmetric pair of cells is one in which the cells are at the same location in the design with interchange of the $0-1$ labelling of the cells and a reversal of both time and cluster order. Formally, the location of the partner cell of a specific cell in a centrosymmetric pair can be obtained by the destination of the cell after reflecting the design schematic along the central horizontal and vertical axes: for a standard stepped wedge over *T* time periods, and $K=T-1$ sequences, the cell in cluster *k* and period *j*, with indices $\left( k,j\right)$, is the centrosymmetric partner of the cell with indices $\left( K+1-k, T+1-j \right)$ [3] (see Fig. 2). In this paper we will obtain progressively reduced designs by removing a centrosymmetric pair of cluster-period cells at each iteration, thus maintaining the centrosymmetry of the reduced designs.

We modify the information content definition from Kasza et al. [3] in two ways: by (1) replacing the numerator with the variance of the treatment effect estimator for designs with a centrosymmetric pair of cells removed rather than a single cell, and (2) replacing the denominator with an expression for $var\hat{(\theta )}$ that accommodates incomplete designs.

Let *A* represent a pair of centrosymmetric cells: $A=\{(k,j), (K+1-k, T+1-j)\}$ for some cluster *k* and period *j*. Suppose that *D* is a skew-symmetric design. Definition 1 of Bowden et al. [9] states that a longitudinal cluster randomised trial design is skew-symmetric if the clusters can be ordered so that $X_{kj}=1-X_{K+1-k,T+1-j}$ or no measurements are taken in both $X_{kj}$ and $X_{K+1-k,T+1-j}$ . Both designs in Fig. 1 are skew-symmetric designs, for example. This design *D* may be a complete stepped wedge design or an incomplete stepped wedge design, where centrosymmetric cell pairs have been removed from a complete stepped wedge design. Then let $var_{D}\hat{(\theta )}$ be the variance of the treatment effect estimator for trial design *D*. We denote the variance of the treatment effect estimator when we delete *A* from *D* as $var_{D[A]}\hat{(\theta )}$. We then define the information content of the cells *A* within the design *D* as:

5 $$\begin{aligned} IC_{D}(A)=var_{D[A]}\hat{(\theta )}/var_{D}\hat{(\theta )} \end{aligned}$$

A derivation of the analytical form of the information content of pairs of cells is provided in Section B of the Additional file 1. Although we will calculate the variances and information content numerically, we show in the derivation of the analytical form that the precision of the reduced design can be represented as the sum of the precision of the previous design and a constant. This means that the information content can be represented as a function of the variance of the previous design and the constant term which is similar to the work in Kasza and Forbes [3].

#### Removal of pairs of cells

To obtain progressively reduced designs, we remove centrosymmetric pairs of cells with low information content in an iterative manner starting from a complete stepped wedge design. For the initial design we calculate the information content for each of the $KT/2 = T(T-1)/2$ centrosymmetric cluster-period cell pairs. The next step is to identify the cell pairs with the lowest information content, and then remove them from the initial design. Where multiple pairs of cells have the same information content, we remove the pair with the smallest cluster and period index (the cell closest to the top-left-hand corner of the design and its centrosymmetric cell counterpart) so that only one pair of cells is removed at each iteration. We then calculate the information content for each of the remaining pairs and remove the pair with the lowest information content for this reduced design. This process continues until the treatment effect cannot be estimated for the reduced design. The algorithm is written out below.

Let $D_{0}$ denote the initial complete design. For the design $D_{l}$ at iteration *l*, $l\ge 1$, let $A_{l}^*$ denote the centrosymmetric pair of cells with the lowest information content corresponding to design $D_{l}$, and then define $D_{l}=D_{l-1}[A_{l-1}^*]$ to be the design at iteration *l* obtained by omitting the cell-pair $A_{l-1}^*$ from design $D_{l-1}$. We then define the information content of a centrosymmetric pair of cells *A* in design $D_{l}$ as the ratio of the variance of the treatment effect estimator for the design $D_{l}[A]$ to the variance for design $D_{l}$: $IC_{D_{l}}(A)=var_{D_{l}[A]}\hat{(\theta )}/var_{D_{l}}\hat{(\theta )}$, where $D_{l}=D_{l-1}[A_{l-1}^*]$ for $l = 1, 2, \dots$ up to a maximum of $T(T-1)/2 -1$. The algorithm proceeds as follows:

1. Calculate the information content of each centrosymmetric pair of cells *A* in design $D_{0}$: $IC_{D_{0}}(A)$.
2. Omit the pair of cells $A_{0}^*$ with the lowest information content. If more than one pair has the same information content, we remove the pair containing the cell with the smallest cluster and period index so that only one pair is removed at each iteration.
3. Generate the reduced design $D_{1}= D_{0}[A_{0}^*]$.

For iterations $l = 2,\dots$ up to a maximum of $T(T-1)/2 -1$:

4 Calculate the information content of each pair of cells *A* in the reduced design $D_{l-1}$: $IC_{D_{l-1}}(A)$.
5 Remove the pair of cells $A_{l-1}^*$ with the lowest information content.
6 Generate the reduced design $D_{l}=D_{l-1}[A_{l-1}^*]$.

Iterate steps $4-6$ until the treatment effect can no longer be estimated. To allow for estimation of the treatment effect, there must be at least one period which contains at least one intervention and at least one control cell. The final design reached by the algorithm may contain more than two cells to allow for this.

#### Relative precision metric

We define a measure of “precision loss” to compare the precision of each reduced design with the precision of the complete design. The precision loss of design $D_{l}$ relative to the complete design $D_{0}$ is defined as:6Higher values correspond to greater loss of precision compared to the complete design, and $0\%$ corresponds to no loss of precision.

## Results

### Illustrative examples

#### The Hill et al. trial

We first obtain progressively reduced designs for a trial configuration motivated by the Hill et al. stepped wedge trial [11] that was explored in Kasza et al. [5]. We consider a simplification of the original trial, with a complete stepped wedge design with 4 clusters and 5 periods, and an equal number of participants per cluster-period (90 participants per cluster-period, the average cluster-period size for this trial). The corresponding trial design schematic is illustrated by the complete design given in Fig. 1. We consider both exchangeable and discrete-time decay correlation structures, and assume the same correlation parameter values as in Kasza et al. [5]: $\rho =0.14$ for the exchangeable structure, and for the discrete-time decay structure, $\rho =0.15$ together with a decay in correlation of $5\%$ per period, corresponding to a CAC of $r=0.95$.

**Fig. 3:**
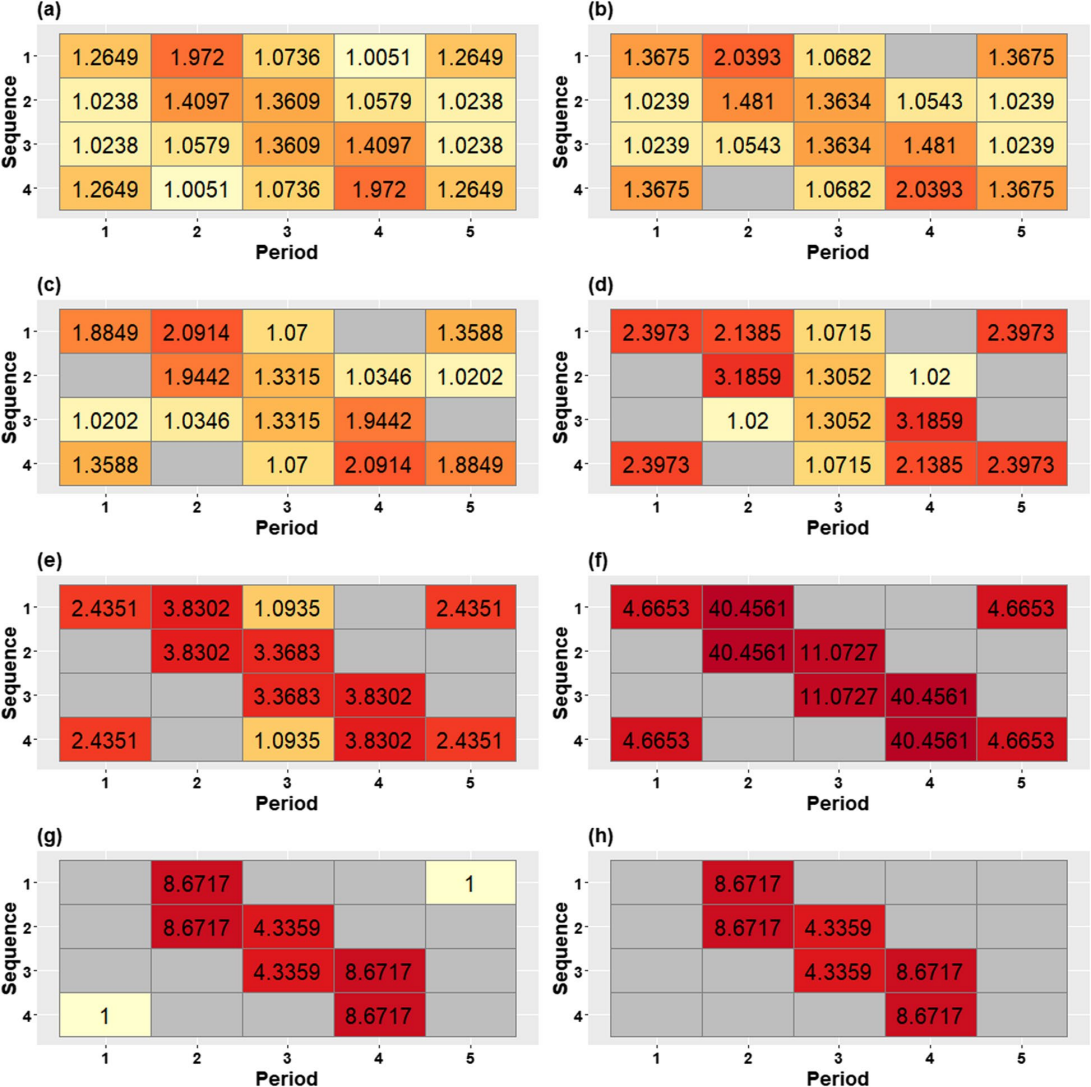
An illustrative example of the changing contributions of different cluster-period cells to estimation of the treatment effect in a stepped wedge design with four clusters (rows in each grid) and five periods (columns), with 90 subjects measured in each cluster-period, under an exchangeable correlation structure with $\rho =0.14$ (and $r=1$). The information content of each cell pair is displayed in each cell, with cell shading also representing the information content, ranging from light yellow (small values) to dark red (large values). The complete stepped wedge design is shown in the top left and the designs become progressively reduced as cluster-period cells are removed from the design, moving from left to right along each row, top row to bottom row

**Fig. 4:**
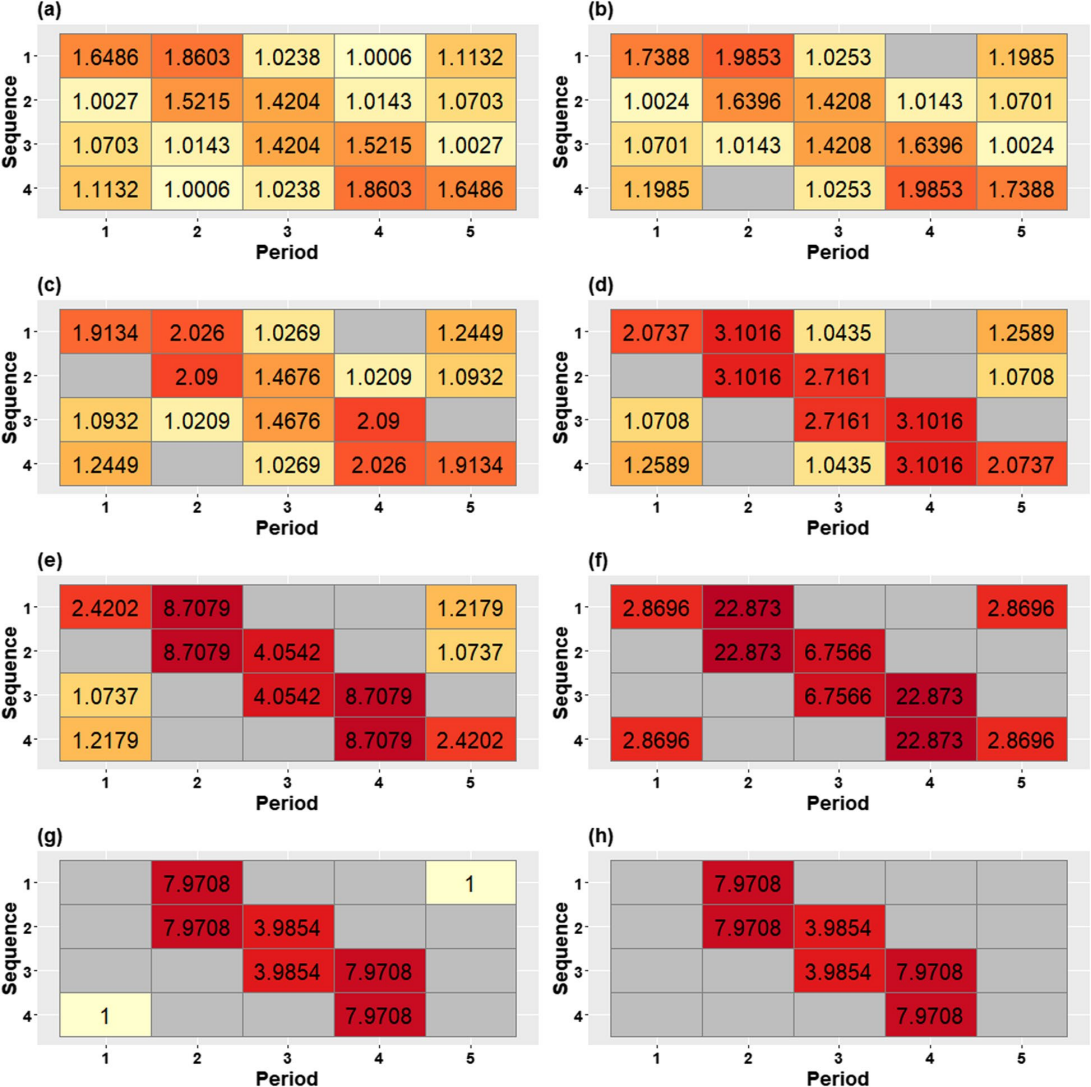
An illustrative example of the changing contributions of different cluster-period cells to estimation of the treatment effect in stepped wedge designs with four clusters (rows in each grid) and five periods (columns), with 90 subjects measured in each cluster-period, under a discrete-time decay correlation structure with $\rho =0.15$ and $r=0.95$. The information content of each cell pair is displayed in each cell, with cell shading also representing the information content, ranging from light yellow (small values) to dark red (large values). The complete stepped wedge design is shown in the top left and the designs become progressively reduced as cluster-period cells are removed from the design, moving from left to right along each row, top row to bottom row

Figures 3(a-h) and 4(a-h) display the progressively reduced designs at each iteration obtained from applying the algorithm in the Removal of pairs of cells subsection to this design, assuming exchangeable correlation and discrete-time decay correlation structures, respectively. The designs in both figures display the information content of each centrosymmetric cell pair through the information content value and cell colour, with darker colours indicating more information-rich cell-pairs. Progressive removal of low information content cell-pairs shows that cluster-period cells distant from the time of the treatment switch are generally removed first. Cells immediately before and after the time of the treatment switch remain until near the end. A comparison between Figs. 3 and 4 shows that the patterns of cell removal are slightly different between the two correlation structures. For the exchangeable correlation structure, information-rich cells are concentrated in the off-diagonal corners of the design in addition to the main diagonal. But, for the discrete-time decay correlation structure, more information is concentrated around the treatment switches while cells away from the main diagonal contain less information. The final design shown in Fig. 3(h) indicates that the middle pair of cells could be deleted. However, after deletion of that pair of cells, the information content of the remaining cells in the design with only four remaining cluster-period cells cannot be calculated.

**Fig. 5:**
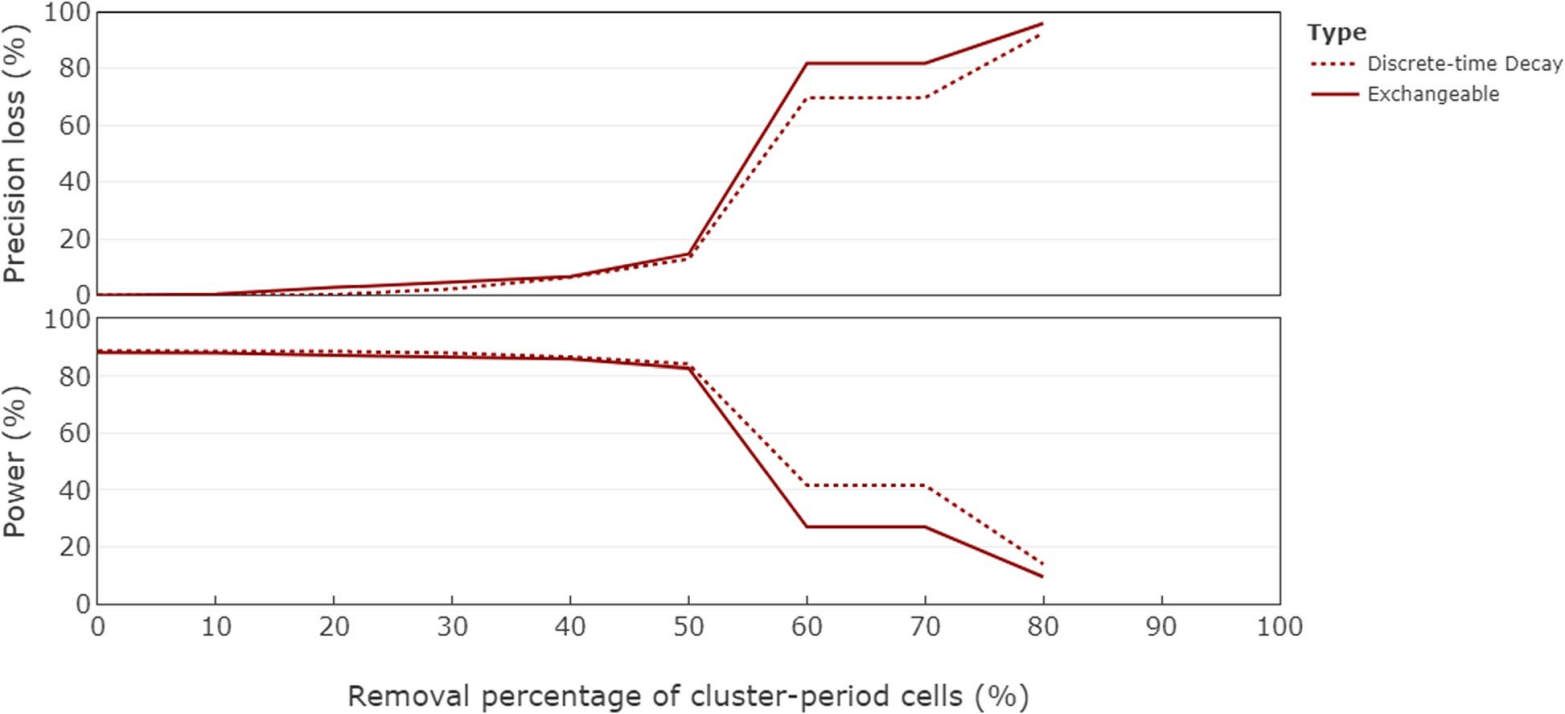
Precision loss (top panel) and power (bottom panel) corresponding to designs obtained through the iterative removal process, indicated by the removal percentage of cluster-period cells, for the exchangeable model (solid line) with a within-period ICC of $\rho =0.14$ and a standardised effect size of 0.25, and the discrete-time decay model (dotted line) with a within-period ICC of $\rho =0.15$ and CAC of $r=0.95$, and a standardised effect size of 0.35

Using the metrics of precision loss and power to assess the range of designs in Figs. 3 and 4, we find that changes in precision and power are non-linear functions of the proportion of cells that have been removed. We consider a standardised effect size of 0.25 when considering a model with an exchangeable correlation structure, and an effect size of 0.35 for the model with a discrete-time decay correlation structure. This implies study power of the complete design of around $90\%$ with a two-sided significance level of 0.05 for each complete design. Figure 5 displays the precision loss (top panel) and power (bottom panel) for the two correlation structures for each of the designs in Figs. 3 and 4 according to the proportion of the total number of cluster-period cells that have been removed. Precision loss increases slightly but remains low until around half of cluster-period cells have been removed under both models; there is a sharp increase in precision loss just after the midway point, with a slightly steeper increase under the exchangeable model than the discrete-time decay model. This jump in precision loss appears to arise from removing cells from the corners, which we refer to as “hot-spot” corners, of the incomplete design when half of the cluster-period cells are removed, with a greater impact for the exchangeable correlation structure than for the discrete-time decay structure. Power, being directly related to the variance of the treatment effect estimator for a particular effect size, displays the same trajectory as precision loss, only mirrored (i.e. increases in the precision loss metric correspond to reductions in power). For the exchangeable model, the complete design has $88.23\%$ power to detect an effect size of 0.25, reducing to $82.83\%$ with a corresponding precision loss of $14.60\%$ for the incomplete design obtained once $50\%$ of the cluster-period cells were removed. This corresponds to the design shown in Fig. 3(f). For the discrete-time decay model, the complete design has $88.78\%$ power to detect a standardised effect size of 0.35, reducing to $84.24\%$ with a corresponding precision loss of $12.84\%$ for the incomplete design obtained once $50\%$ of the cluster-period cells were removed, corresponding to the design shown in Fig. 4(f).

#### A larger design

**Fig. 6:**
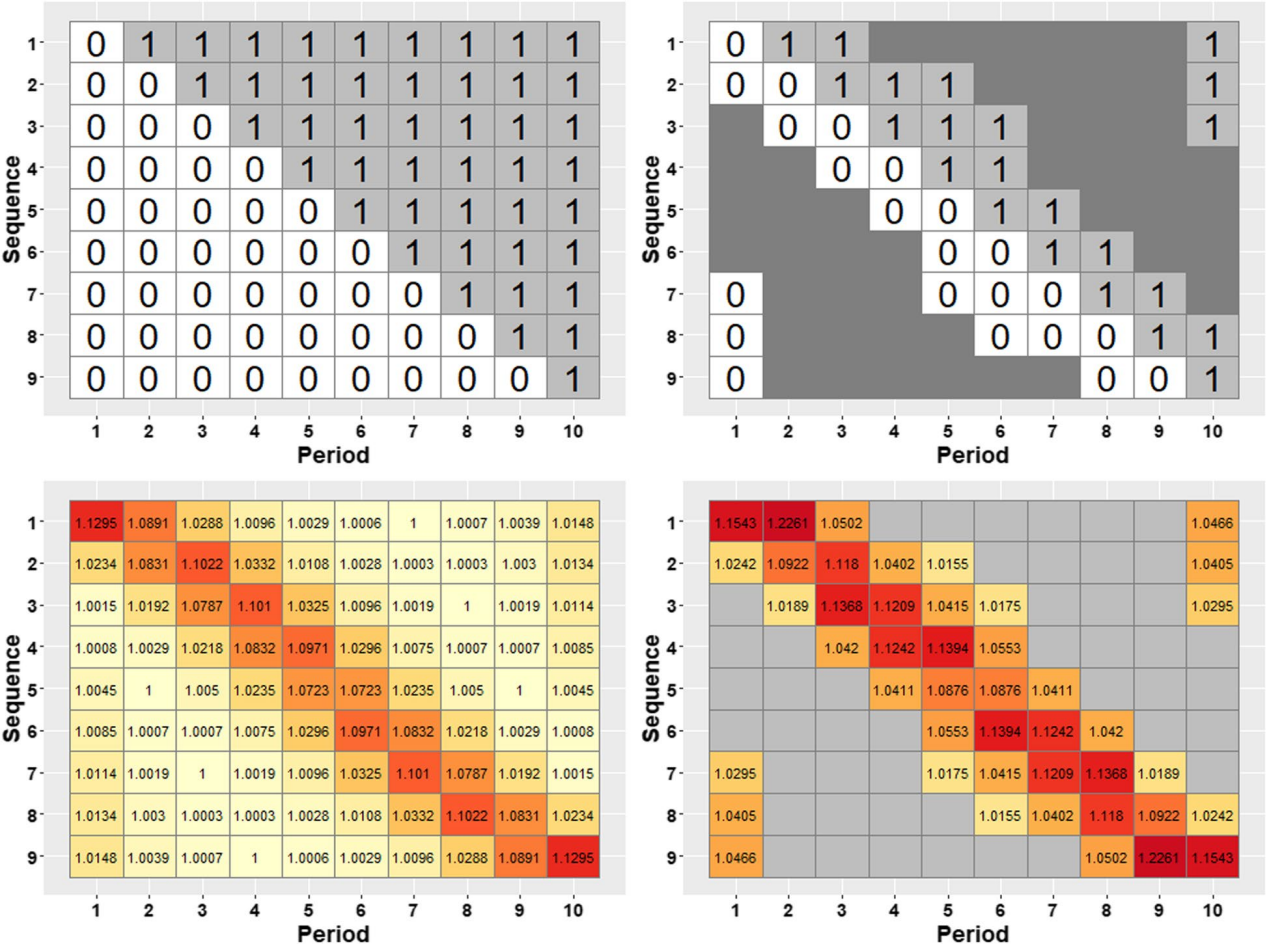
Top-left: Design schematic of a 10-period complete stepped wedge design. Top-right: Design schematic of a 10-period incomplete stepped wedge design when $51.1\%$ of cluster-period cells have been removed. Bottom-left: Information content of the cells in a complete stepped wedge design with 50 subjects measured in each cluster-period, assuming a discrete-time decay model with $\rho =0.05$ and $r=0.95$, with $90.18\%$ power to detect a standardised effect size of 0.2. Bottom-right: Information content of the cells in the incomplete stepped wedge design depicted in the top-right panel, for the same configuration as the complete design when $51.11\%$ cluster-period cells removed. The corresponding power is $88.35\%$ and $6.02\%$ precision is lost

In this subsection we consider a design with a larger number of periods to further illustrate the algorithm: a stepped wedge design with 9 clusters, 10 periods, and 50 participants per cluster-period. We consider the discrete-time decay correlation structure, assuming a within-period ICC of $\rho =0.05$ with a decay in correlation of $5\%$ per period, corresponding to a CAC of $r=0.95$. The schematic of the complete stepped wedge design is shown in the top-left panel of Fig. 6, with the information content of pairs of cluster-period cells of the complete design illustrated below. The right-hand side of Fig. 6 displays the incomplete design after approximately $50\%$ of cluster-period cells have been removed, with the information content of cluster-period cell pairs shown below. This incomplete design appears similar to a “staircase design” where clusters contribute measurements immediately before and after the treatment switch, but with some additional measurements in the first and final periods of the design, and different sequences contain different numbers of control and intervention periods. As in the The Hill et al. trial subsection, information is once again concentrated near the time of the treatment switch. The complete set of reduced designs commencing with the complete design and terminating with the minimally viable design is displayed in the Additional file 1 (Fig. C1).

**Fig. 7:**
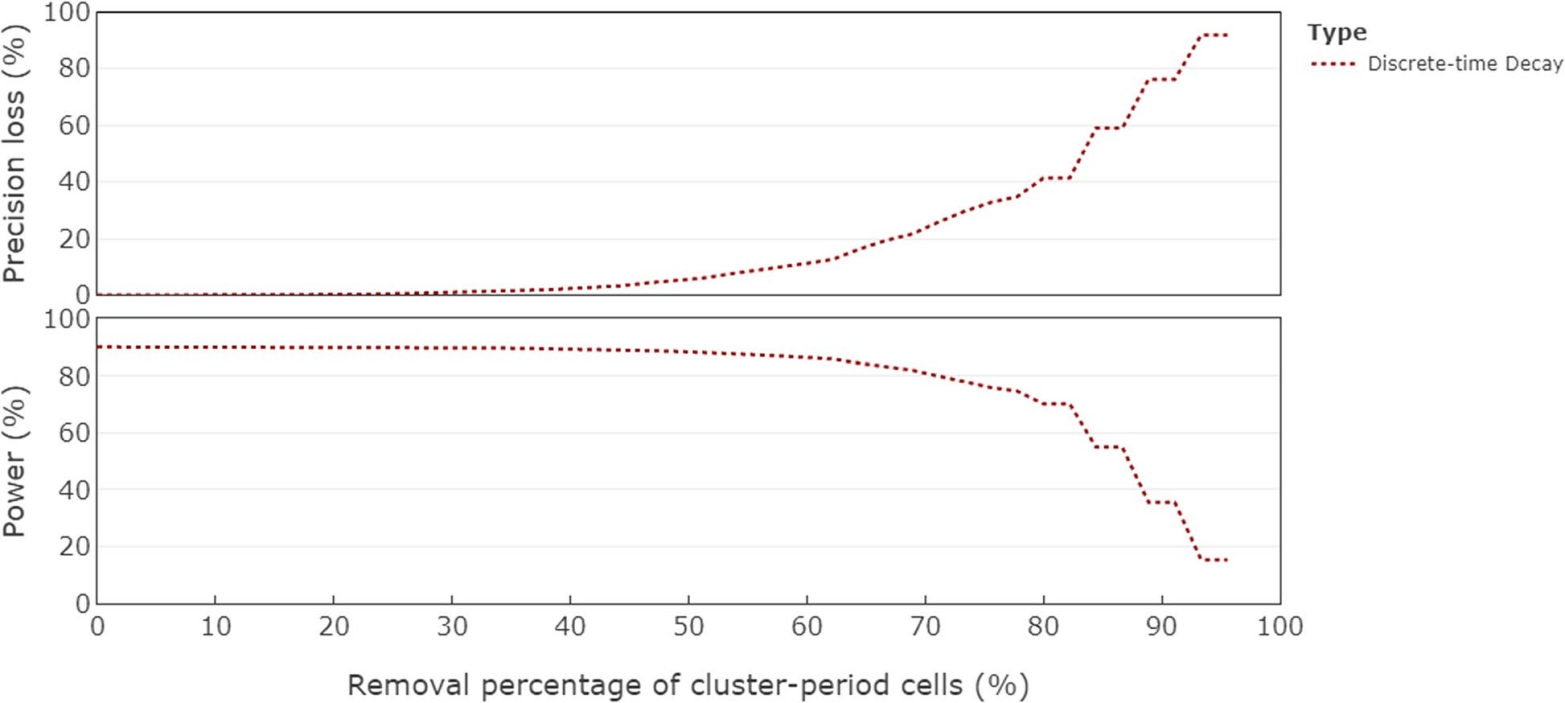
Precision loss (top panel) and power (bottom panel) corresponding to designs obtained through the iterative removal process, indicated by the removal percentage of cluster-period cells, for the discrete-time decay model with $\rho =0.05$ and $r=0.95$, and a standardised effect size of 0.2

Figure 7 displays the precision loss (top panel) and power (bottom panel) for the discrete-time decay correlation structure of the series of progressively reduced designs in the Additional file 1 (Fig. C1) according to the proportion of the total number of cluster-period cells that have been removed. We consider a standardised effect size of 0.2, yielding $90.2\%$ power for the complete design. The reduced design at the midway point, i.e. when approximately $50\%$ of cluster-period cells are removed, is only $6\%$ less efficient than the complete design and the power is only $2\%$ lower than for the complete design.

Researchers can explore progressively reduced designs for this and other trial settings, along with plots of study power and precision loss, with our web app available at https://monash-biostat.shinyapps.io/iterativeinfcontent/.

### More general results

To assess broad patterns of precision loss across a range of trial configuration parameters likely to be seen in practice, we explore the precision loss of progressively reduced designs for several combinations of correlation and trial configuration parameters (See Table 1). We consider stepped wedge designs with 5 and 10 periods, with 10 or 100 participants per cluster-period, with within-period ICC values of 0.01, 0.05, and 0.15, with cluster autocorrelation values of 1, 0.95, and 0.8. We apply the iterative removal algorithm to each of these sets of configurations, and calculate the precision loss for the designs obtained by removing $20\%$, $50\%$ and $80\%$ of the cluster-period cells. For the 5-period designs, this corresponded to designs with $80\%$, $50\%$ and $20\%$ of cells remaining; for the 10-period designs, this corresponded to designs with $80\%$, $48.89\%$ and $20\%$ of cells remaining.

**Table 1:** The parameters used for generating Fig. 8

Parameters	Values
Number of periods, *T* (Number of clusters, *K*)	5(4) , 10(9)
Number of subjects per cluster-period, *m*	10, 100
Intracluster correlation (ICC), $\rho$	0.01, 0.05, 0.15
Cluster autocorrelation, *r* (Correlation structure)	1 (Exchangeable)
	0.95, 0.8 (Discrete-time decay)
Removal percentage of cluster-period cells	$20\%$, $50\%$, $80\%$

**Fig. 8:**
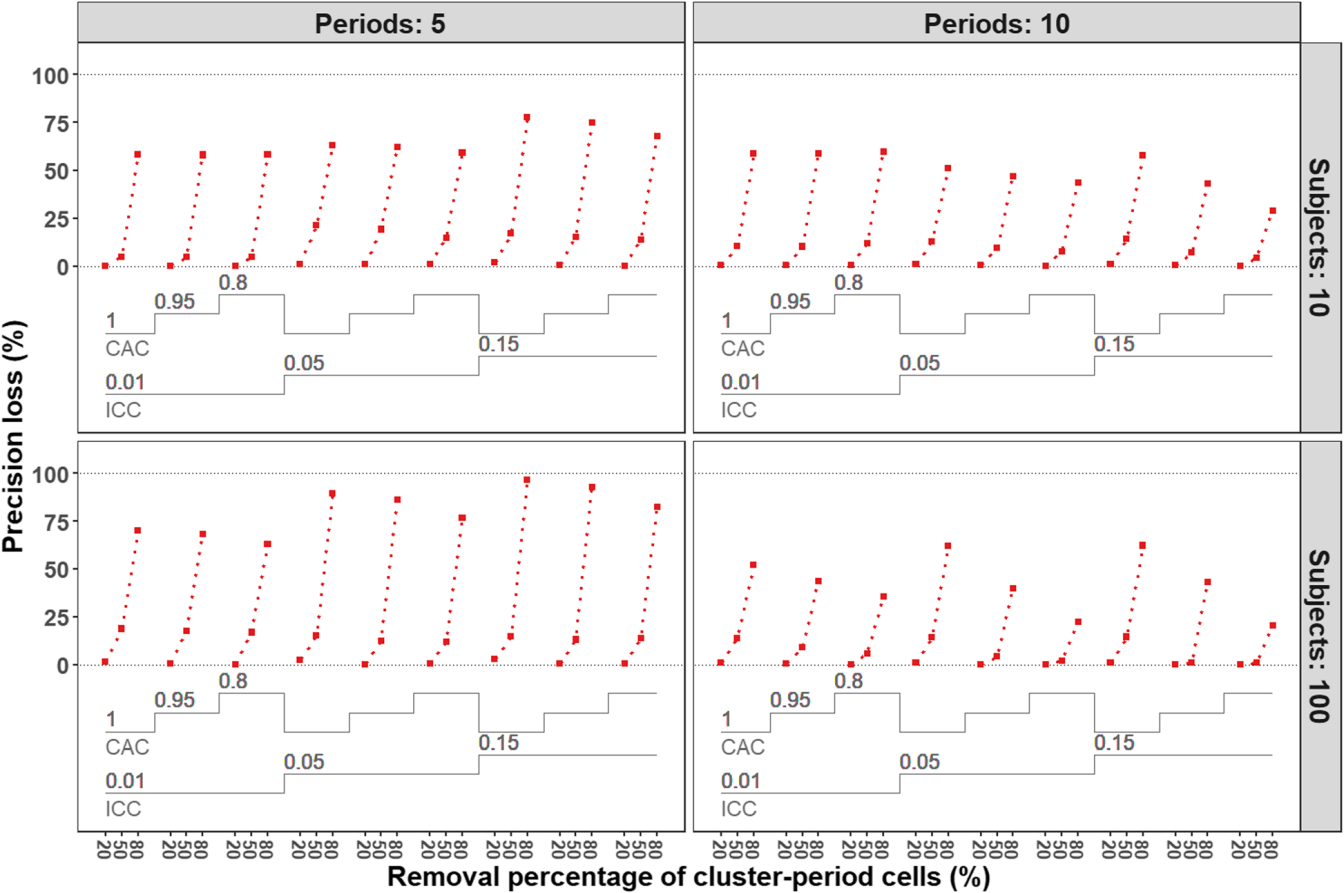
Precision loss for incomplete designs with three different percentages of cluster-period cells removed ($20\%$, $50\%$, $80\%$). Each panel corresponds to a different combination of periods and subjects per cluster-period. They consist of three prespecified correlation parameters: within-period ICC $\rho$ $\left( 0.01, 0.05, 0.15\right)$ and cluster autocorrelation *r* $\left( 1, 0.95,0.8\right)$. In total there are 36 $\left( 2 \times 2 \times 3 \times 3\right)$ different trial configurations

Figure 8 displays the precision loss for the incomplete designs with the predefined removal percentages across all combinations of the trial configurations. The broad patterns of precision loss are fairly consistent across all the designs considered: designs with $20\%$ of cluster-period cells removed generally have very little precision loss; designs with around $50\%$ of cluster-period cells removed have slightly higher loss of precision, but not exceeding $25\%$; and designs with $80\%$ of cluster-period cells removed have much higher loss of precision. In general, less precision is lost at the predefined checkpoints in the removal process for designs with more periods compared to designs with fewer periods, for equivalent correlation parameter values. Across all trial configurations, precision loss tends to be slightly lower under the discrete-time decay correlation structure $(r=0.95$ or 0.8) than the exchangeable correlation structure $\left( r=1\right)$. When there are a large number of participants and a small number of periods (bottom-left panel), more precision is lost for various correlation parameters as compared to the same number of periods but with a small number of participants (top-left panel). When $20\%$ of cluster-period cells are removed, the reduced designs have almost the same precision as the complete designs. The minimum and maximum precision loss when $20\%$ of cells are removed are $0.01\%$ and $2.86\%$, respectively and when approximately $50\%$ of cells are removed are $0.99\%$ and $21.21\%$, respectively. This indicates that incomplete designs are likely to provide useful alternatives to complete stepped wedge designs across a broad range of designs and intracluster correlation structures.

## Discussion

In this paper, we have considered an iterative approach to obtaining progressively reduced designs by removing pairs of cells with low information content for standard stepped wedge designs with repeated cross-sectional sampling. Where previous work focused on the information content of individual cells within a complete stepped wedge, we continually updated the information content of cell *pairs* across a range of designs, from the complete design to a minimally viable design. Our findings show that for many trial configurations, incomplete stepped wedge designs with up to $50\%$ of cluster-period cells removed are still nearly as efficient as the complete stepped wedge, with less than $25\%$ precision loss and typically only a small reduction in study power. Removing hot-spot corners generally has a larger impact for the exchangeable intracluster correlation structure than for the discrete-time decay correlation structure. This is due to the reduction in the similarity of observations from the same cluster as the time between their measurements increases when the discrete-time decay correlation structure is assumed: measurements in these corners do not offer as much information about the treatment effect when these subjects’ outcomes are less similar to subjects’ outcomes near the time of the treatment switch in that cluster.

In Section C of Additional file 1 we provide an additional example where both the within-period ICC and cluster-period size are small: $\rho = 0.01$ and $m=10$. As illustrated in Fig. C2(x) for this setting, the incomplete stepped wedge design where hot-spot corners are not present, is still able to provide sufficient power and high precision (power reduction: $3.26\%$, precision loss: $10.20\%$). For this value of *m* and $\rho$, the mixed model uses mainly vertical comparisons rather than horizontal [12]. Therefore, the first and last period contribute nothing to these comparisons because they are all 0’s or all 1’s. There is still some contribution from horizontal comparisons so the cells just before and after the intervention switch are still useful horizontally. For this design, measurements in the first and final periods are removed during the early iterations of the algorithm. The intuition for this finding arises from the work of Matthews and Forbes [12] in which settings with a small cluster-period size and small within-period ICC lead to the treatment effect estimator being dominated by vertical comparisons. With the first and final periods containing all control and all intervention conditions, respectively, there is no information within these periods upon which to estimate the treatment effect, and correspondingly these cells are removed early in the iterative removal process (Fig. C2).

Our work indicates that designs which resemble staircase designs, where the number of pre- and post-switch measurement periods differ across the sequences of the design, can be highly efficient. Further, depending on the design parameters, these reduced designs may include measurement periods in the first and final periods of the design. That is, were such designs adopted, some clusters would need to provide measurements at the beginning and end of the trial, without needing to provide measurements in the middle of the trial. The feasibility and usefulness of such designs in practice would require discussion with trialists in the specific subject matter context. This work indicates that staircase designs may often be efficient alternatives to complete stepped wedge designs, but more research is required to investigate the statistical properties of these designs. For example, we are currently investigating the ways in which observations within a complete stepped wedge design could be re-arranged or re-distributed to provide staircase designs with as much power as the complete design.

The patterns of information content we obtained across a range of incomplete designs are generally consistent with those found previously for complete stepped wedge designs [3–5]: cells closest to the time of the treatment switches contain by far the most information for estimation of the treatment effect and the off-diagonal corners may also contribute a great deal of information. Moreover, we found that incomplete designs such as those depicted in Figs. 3(f) and 4(f) were highly efficient: these designs resemble Design B in Section 4 of Kasza et al. [5], chosen for its retention of cells with the highest information content under their definition. Another algorithmic search for selecting an efficient design has previously been used to remove two participants at each iteration using iterative improvements with continuous recruitment; i.e. time was considered a continuous phenomenon in that paper [8]. Although none of the reduced designs in that paper were exact staircase designs (where each cluster contributes measurements in a restricted number of consecutive pre- and post-switch periods) [3], in certain scenarios that algorithm found designs that closely approximated staircase designs. Our work can be considered as a discrete-time version of Hooper et al. [8]: our algorithm searches for cells with the lowest information content at each iteration while considering time as a discrete phenomenon (as is common in the design and analysis of stepped wedge trials).

While broad patterns of information content appear to be fairly consistent across a range of trial configuration parameters, we provide an online app at https://monash-biostat.shinyapps.io/iterativeinfcontent/ so that readers can explore incomplete designs for user-defined trial configurations. This app enables trialists to specify their desired trial configurations including the number of periods, the number of participants in each cluster-period, within-period intracluster correlation ICC, cluster autocorrelation CAC, type of correlation structure, and the effect size of interest. Selecting ‘Yes’ in the ‘allow for decay correlation’ option and specifying a CAC lower than 1 in the app enables a discrete-time decay correlation structure. However, block-exchangeable correlation structures can also be accommodated, by selecting ‘No’ in the ‘allow for decay correlation’ option, and providing a CAC lower than 1. Finally, to choose an exchangeable correlation structure, again select ‘No’ in the ‘allow for decay correlation’ option while setting the CAC to 1.

There are a number of aspects of this work that can be extended. Our results pertain to settings where continuous outcomes are analysed with linear mixed models, but settings with binary outcomes are also common. While it is possible that different patterns might emerge when considering binary outcomes, given the similarity of the results in Li et al. [13] for binary outcomes and marginal models with those of Kasza and Forbes [3] for linear mixed models, we would expect this similarity to carry through to incomplete designs. We also assumed equal cluster-period sizes in this paper, but further work could consider unequal cluster-period sizes arising from different numbers of individuals recruited per cluster-period, or with cohort sampling structures with dropout or loss to follow-up. Our work could also be extended to consider designs with transition periods, meaning no data collection in the period(s) just prior to commencement of the intervention. Additionally, we assumed no treatment effect heterogeneity is present, otherwise the centrosymmetric properties would not hold for the information content of cells [4], and therefore the extent of the impact of treatment effect heterogeneity requires further research. Finally, in further work we intend to evaluate design precision while incorporating the associated costs as considered by Grantham et al. [14].

## Conclusions

In summary, obtaining incomplete designs guided by the information content of pairs of cluster-period cells ensures that any measurements taken are going to be highly informative for estimating the treatment effect. We have shown that certain incomplete stepped wedge designs with measurements concentrated around the main diagonal (and possibly in the hot-spot corners) may result in only a small precision loss relative to the complete stepped wedge design and hence may be nearly as powerful as the full stepped wedge design.

## Supplementary Information

**Additional file 1.**

## Data Availability

Data sharing is not applicable to this article as no new data were created or analyzed in this study. Project code is available at https://github.com/EhsanRD/Iterative-Removals-SW.

## Abbreviations

ICC: Intracluster correlation
CAC: Cluster autocorrelation
IC: Information content
