# Supplementary material for: The impact of iterative removal of low-information cluster-period cells from a stepped wedge design
Source: BMC Med Res Methodol. 2023 Jul 6;23:160. doi: 10.1186/s12874-023-01969-7 (PMC10324156; doi:10.1186/s12874-023-01969-7)
Supplement: Supplementary file 1 — Additional file 1. [file 12874_2023_1969_MOESM1_ESM.pdf]

Additional file 1: Derivations and additional figures

The impact of iterative removal of low-information cluster-period cells from a stepped wedge design

Ehsan Rezaei-Darzi, Kelsey L Grantham, Andrew B Forbes, Jessica Kasza

A The general variance expression for incomplete designs

We re-state the following general model for a cross-sectional longitudinal cluster randomised trial with a continuous outcome Y_{kji} for subject $i = 1, \ldots, m$ in time period $j = 1, \ldots, T$ in cluster $k = 1, \ldots, K$ as in Kasza and Forbes [1]:

$$Y_{kji} = \mu + \beta_j + X_{kj}\theta + \gamma_{kj} + \epsilon_{kji}, \quad \gamma_k \sim N_{T_k}\left(0, \mathbf{V}_{\gamma k}\right), \quad \epsilon_{kji} \sim N\left(0, \sigma_\epsilon^2\right)$$

where T_k is the number of measurement periods in cluster k, and T is the total number of periods in the complete design.

Collapsing the above model to cluster-period means, $\bar{Y}_{kj} = \frac{1}{m}\sum_{i=1}^{m} Y_{kji}$, gives

$$\bar{Y}_{kj} = \mu + \beta_j + X_{kj}\theta + \gamma_{kj} + \epsilon_{kj}, \quad \gamma_k \sim N_{T_k}\left(0, \mathbf{V}_{\gamma k}\right), \quad \epsilon_{kj} \sim N\left(0, \frac{\sigma_\epsilon^2}{m}\right)$$

Letting $\bar{\mathbf{Y}}_k = \left(\bar{Y}_{k1}, \ldots, \bar{Y}_{kT}\right)^T$, and using $\mathbf{V}_k$ to denote the covariance matrix of $\bar{\mathbf{Y}}_k$, $cov(\bar{\mathbf{Y}}_k) = \mathbf{V}_{\gamma k} + \frac{\sigma_\epsilon^2}{m}\mathbf{I} = \mathbf{V}_k$, where $\mathbf{V}_k$ has dimension $T_k \times T_k$. Letting $\bar{\mathbf{Y}} = \left(\bar{\mathbf{Y}}_1^T, \ldots, \bar{\mathbf{Y}}_K^T\right)^T$, then the variance matrix $\mathbf{V}$ of $\bar{\mathbf{Y}}$ is block-diagonal with each of the $\mathbf{V}_k$s along the diagonal, yielding a $\left(\sum_{k=1}^{K} T_k\right) \times \left(\sum_{k=1}^{K} T_k\right)$ -dimensional matrix.

Re-parameterising μ and the time effects as $\beta_1, \ldots, \beta_T$ and writing the equation in vector form gives

$$\bar{\mathbf{Y}} = \mathbf{Z}\begin{bmatrix} \beta_1 \\ \vdots \\ \beta_T \\ \theta \end{bmatrix} + \begin{bmatrix} \gamma_1 \\ \vdots \\ \gamma_K \end{bmatrix} + \epsilon.$$

The usual estimate of the parameter vector $\eta = (\beta_1, \ldots, \beta_T, \theta)^T$ is then given by $\hat{\eta} = \left(\mathbf{Z}^T\mathbf{V}^{-1}\mathbf{Z}\right)^{-1}\mathbf{Z}^T\mathbf{V}^{-1}\bar{\mathbf{Y}}$ with covariance matrix $cov(\hat{\eta}) = \left(\mathbf{Z}^T\mathbf{V}^{-1}\mathbf{Z}\right)^{-1}$. The design matrix $\mathbf{Z}$ can be written as

$$\mathbf{Z} = \begin{bmatrix} \mathbf{Z}_1 & \mathbf{X}_1 \\ \mathbf{Z}_2 & \mathbf{X}_2 \\ \vdots & \vdots \\ \mathbf{Z}_K & \mathbf{X}_K \end{bmatrix}$$

where $\mathbf{Z}_k$ has dimension $T_k \times T$, and $\mathbf{X}_k$ has dimension $T_k \times 1$.

We are interested in obtaining an expression for the variance of the treatment effect estimator,

$var(\hat{\theta})$, the $(T+1)\times(T+1)$th entry of $(\mathbf{Z}^T\mathbf{V}^{-1}\mathbf{Z})^{-1}$. Then

$$\mathbf{Z}^T\mathbf{V}^{-1}\mathbf{Z} =$$

$$\begin{bmatrix} \mathbf{Z}_1^T & \mathbf{Z}_2^T & \dots & \mathbf{Z}_K^T \\ \mathbf{X}_1^T & \mathbf{X}_2^T & \dots & \mathbf{X}_K^T \end{bmatrix} \begin{bmatrix} \mathbf{V}_1^{-1} & & & \\ & \mathbf{V}_2^{-1} & & \\ & & \ddots & \\ & & & \mathbf{V}_K^{-1} \end{bmatrix} \begin{bmatrix} \mathbf{Z}_1 & \mathbf{X}_1 \\ \mathbf{Z}_2 & \mathbf{X}_2 \\ \vdots & \vdots \\ \mathbf{Z}_K & \mathbf{X}_K \end{bmatrix}$$

$$= \begin{bmatrix} \mathbf{Z}_1^T\mathbf{V}_1^{-1} & \mathbf{Z}_2^T\mathbf{V}_2^{-1} & \dots & \mathbf{Z}_K^T\mathbf{V}_K^{-1} \\ \mathbf{X}_1^T\mathbf{V}_1^{-1} & \mathbf{X}_2^T\mathbf{V}_2^{-1} & \dots & \mathbf{X}_K^T\mathbf{V}_K^{-1} \end{bmatrix} \begin{bmatrix} \mathbf{Z}_1 & \mathbf{X}_1 \\ \mathbf{Z}_2 & \mathbf{X}_2 \\ \vdots & \vdots \\ \mathbf{Z}_K & \mathbf{X}_K \end{bmatrix}$$

$$= \begin{bmatrix} \mathbf{Z}_1^T\mathbf{V}_1^{-1}\mathbf{Z}_1 + \mathbf{Z}_2^T\mathbf{V}_2^{-1}\mathbf{Z}_2 + \dots + \mathbf{Z}_K^T\mathbf{V}_K^{-1}\mathbf{Z}_K & \mathbf{Z}_1^T\mathbf{V}_1^{-1}\mathbf{X}_1 + \mathbf{Z}_2^T\mathbf{V}_2^{-1}\mathbf{X}_2 + \dots + \mathbf{Z}_K^T\mathbf{V}_K^{-1}\mathbf{X}_K \\ \mathbf{X}_1^T\mathbf{V}_1^{-1}\mathbf{Z}_1 + \mathbf{X}_2^T\mathbf{V}_2^{-1}\mathbf{Z}_2 + \dots + \mathbf{X}_K^T\mathbf{V}_K^{-1}\mathbf{Z}_K & \mathbf{X}_1^T\mathbf{V}_1^{-1}\mathbf{X}_1 + \mathbf{X}_2^T\mathbf{V}_2^{-1}\mathbf{X}_2 + \dots + \mathbf{X}_K^T\mathbf{V}_K^{-1}\mathbf{X}_K \end{bmatrix}$$

$$= \begin{bmatrix} \sum_{k=1}^K \mathbf{Z}_k^T\mathbf{V}_k^{-1}\mathbf{Z}_k & \sum_{k=1}^K \mathbf{Z}_k^T\mathbf{V}_k^{-1}\mathbf{X}_k \\ \sum_{k=1}^K \mathbf{X}_k^T\mathbf{V}_k^{-1}\mathbf{Z}_k & \sum_{k=1}^K \mathbf{X}_k^T\mathbf{V}_k^{-1}\mathbf{X}_k \end{bmatrix}$$

$$= \begin{bmatrix} \sum_{k=1}^K \mathbf{Z}_k^T\mathbf{V}_k^{-1}\mathbf{Z}_k & \sum_{k=1}^K \mathbf{Z}_k^T\mathbf{V}_k^{-1}\mathbf{X}_k \\ \left(\sum_{k=1}^K \mathbf{Z}_k^T\mathbf{V}_k^{-1}\mathbf{X}_k\right)^T & \sum_{k=1}^K \mathbf{X}_k^T\mathbf{V}_k^{-1}\mathbf{X}_k \end{bmatrix} = \begin{bmatrix} A_{11} & A_{12} \\ A_{21} & A_{22} \end{bmatrix}$$

Note: The $\mathbf{Z}_k$, $\mathbf{V}_k$, and $\mathbf{X}_k$ matrices may have different dimensions across the K clusters due to incompleteness, but these matrices will be conformable for a particular cluster k.

The $(T+1)\times(T+1)$th entry of $(\mathbf{Z}^T\mathbf{V}^{-1}\mathbf{Z})^{-1}$ is given by $\left(A_{22} - A_{21}A_{11}^{-1}A_{12}\right)^{-1}$. We can then write:

$$var(\hat{\theta}) = \left\{ \sum_{k=1}^K \mathbf{X}_k^T\mathbf{V}_k^{-1}\mathbf{X}_k - \left(\sum_{k=1}^K \mathbf{Z}_k^T\mathbf{V}_k^{-1}\mathbf{X}_k\right)^T \times \left(\sum_{k=1}^K \mathbf{Z}_k^T\mathbf{V}_k^{-1}\mathbf{Z}_k\right)^{-1} \left(\sum_{k=1}^K \mathbf{Z}_k^T\mathbf{V}_k^{-1}\mathbf{X}_k\right) \right\}^{-1}.$$

B The information content of pairs of cells for incomplete designs

In this section we extend the derivation of the information content of a general number of cells for a complete design in Web Appendix B.1 of Kasza and Forbes (2019) to obtain an expression for the information content of centrosymmetric pairs of cells of a potentially incomplete design. This design has T periods and cluster k, $k = 1, \dots, K$, has T_k periods of measurements.

The information content expression follows from the expression for $var(\hat{\theta})_{[o]}$, which represents the variance of the treatment effect estimator for the remaining cells once a pair of cells, denoted by o, are removed. We will work with a rearrangement of the cell means $\bar{\mathbf{Y}}$ where the observations corresponding to the omitted pair of cells appear first, in cluster order, followed by the observations corresponding to the included cells (grouped by cluster).

Then the covariance matrix of $\bar{\mathbf{Y}}$ can be decomposed as

$$\mathbf{V} = \begin{pmatrix} \mathbf{V}_o & \mathbf{W} \\ \mathbf{W}^T & \mathbf{V}_{[o]} \end{pmatrix}$$

where $\mathbf{V}_o$ is of dimension 2×2, $\mathbf{V}_{[o]}$ is of dimension $\left(\sum_{k=1}^K T_k - 2\right) \times \left(\sum_{k=1}^K T_k - 2\right)$, and W is of dimension $2 \times \left(\sum_{k=1}^K T_k - 2\right)$. Since the omitted and included cell means are organised by cluster, $\mathbf{V}_o$ has a block diagonal structure, with blocks given by $\mathbf{V}_{m_k}$, where m_k is the number of cells omitted from each cluster k, and $\mathbf{V}_{m_k}$ is either the variance of the omitted cell or covariance matrix of the omitted pair of cells from cluster k. Note that since we are considering removing two cells at a time, m_k will be 0 if no cells are removed from cluster k, 1 if a single cell is removed, or 2 if the pair of cells belongs to cluster k. The matrix $\mathbf{V}_{[o]}$ also has a block diagonal structure, with blocks $\mathbf{V}_{[m_k]}$: the covariance matrix of the included cells of cluster k. The matrix W can be decomposed as

$$\mathbf{W} = \begin{pmatrix} \mathbf{C}_{m_1} \\ \vdots \\ \mathbf{C}_{m_K} \end{pmatrix}$$

where each $\mathbf{C}_{m_k}$, $k = 1, \ldots, K$, is of dimension $m_k \times \left(\sum_{k=1}^K T_k - 2\right)$, and contains the covariances between the omitted observation(s) in cluster k and all included observations. Due to independence between clusters, the only non-zero entries of $\mathbf{C}_{m_k}$ will correspond to covariances between omitted and included observations in the same cluster: $\mathbf{C}_{m_k}$ will contain $T_k - m_k$ non-zero columns. We denote those non-zero columns as $\mathbf{C}_{m_k}^{\neq 0}$.

The inverse of the covariance matrix, $\mathbf{V}^{-1}$, is then given by

$$\mathbf{V}^{-1} = \begin{pmatrix} \left(\mathbf{V}_o - \mathbf{W}\mathbf{V}_{[o]}^{-1}\mathbf{W}^T\right)^{-1} & -\left(\mathbf{V}_o - \mathbf{W}\mathbf{V}_{[o]}^{-1}\mathbf{W}^T\right)^{-1}\mathbf{W}\mathbf{V}_{[o]}^{-1} \\ -\mathbf{V}_{[o]}^{-1}\mathbf{W}^T\left(\mathbf{V}_o - \mathbf{W}\mathbf{V}_{[o]}^{-1}\mathbf{W}^T\right)^{-1} & \mathbf{V}_{[o]}^{-1} + \mathbf{V}_{[o]}^{-1}\mathbf{W}^T\left(\mathbf{V}_o - \mathbf{W}\mathbf{V}_{[o]}^{-1}\mathbf{W}^T\right)^{-1}\mathbf{W}\mathbf{V}_{[o]}^{-1} \end{pmatrix}.$$

The design matrix $\mathbf{Z}$ can be decomposed as

$$\mathbf{Z} = \begin{pmatrix} \mathbf{Z}_o \\ \mathbf{Z}_{[o]} \end{pmatrix}$$

where $\mathbf{Z}_o$ is the $2 \times (T+1)$ sub-matrix of $\mathbf{Z}$ corresponding to the omitted cells, and $\mathbf{Z}_{[o]}$ is the $(TK - 2) \times (T+1)$ sub-matrix of $\mathbf{Z}$ corresponding to the included cells.

Standard matrix algebra gives

$$\mathbf{Z}^T\mathbf{V}^{-1}\mathbf{Z} = \mathbf{Z}_{[o]}^T\mathbf{V}_{[o]}^{-1}\mathbf{Z}_{[o]} + \left(\mathbf{Z}_o^T - \mathbf{Z}_{[o]}^T\mathbf{V}_{[o]}^{-1}\mathbf{W}^T\right)\left(\mathbf{V}_o - \mathbf{W}\mathbf{V}_{[o]}^{-1}\mathbf{W}^T\right)^{-1}\left(\mathbf{Z}_o^T - \mathbf{Z}_{[o]}^T\mathbf{V}_{[o]}^{-1}\mathbf{W}^T\right)^T$$

so that

$$\mathbf{Z}_{[o]}^T\mathbf{V}_{[o]}^{-1}\mathbf{Z}_{[o]} = \mathbf{Z}^T\mathbf{V}^{-1}\mathbf{Z} - \tilde{\mathbf{Z}}_o\mathbf{D}^{-1}\tilde{\mathbf{Z}}_o^T$$

where $\tilde{\mathbf{Z}}_o = \mathbf{Z}_o^T - \mathbf{Z}_{[o]}^T\mathbf{V}_{[o]}^{-1}\mathbf{W}^T$ and $\mathbf{D} = \mathbf{V}_o - \mathbf{W}\mathbf{V}_{[o]}^{-1}\mathbf{W}^T$.

Thus $var(\hat{\theta})_{[o]}$ is given by the $(T+1) \times (T+1)^{th}$ entry of

$$\left(\mathbf{Z}_{[o]}^T\mathbf{V}_{[o]}^{-1}\mathbf{Z}_{[o]}\right)^{-1} = \left(\mathbf{Z}^T\mathbf{V}^{-1}\mathbf{Z} - \tilde{\mathbf{Z}}_o\mathbf{D}^{-1}\tilde{\mathbf{Z}}_o^T\right)^{-1}.$$

Since V_o and $V_{[o]}$ are block diagonal matrices, the matrix D will have the form:

$$\mathbf{D} = \begin{pmatrix} \mathbf{V}_{m_1} - \mathbf{C}^{\neq 0}_{m_1}\mathbf{V}^{-1}_{[m_1]}\mathbf{C}^{\neq 0T}_{m_1} & \dots & 0 \\ \vdots & \ddots & \vdots \\ 0 & \dots & \mathbf{V}_{m_K} - \mathbf{C}^{\neq 0}_{m_K}\mathbf{V}^{-1}_{[m_K]}\mathbf{C}^{\neq 0T}_{m_K} \end{pmatrix}$$

where again $\mathbf{C}^{\neq 0}_{m_k}$ contains only the non-zero columns of $\mathbf{C}_{m_k}$. We can write the matrix $\tilde{\mathbf{Z}}_o$ as

$$\tilde{\mathbf{Z}}_o = \begin{pmatrix} \tilde{\mathbf{T}}_{m_1} & \dots & \tilde{\mathbf{T}}_{m_K} \\ \tilde{\mathbf{X}}_{m_1} & \dots & \tilde{\mathbf{X}}_{m_K} \end{pmatrix}$$

where $\tilde{\mathbf{T}}_{m_k}$ is of dimension $T \times m_k$ (or m_k column(s) vector of length T) and represents the modified time effect(s) in cluster k and $\tilde{\mathbf{X}}_{m_k}$ is of dimension $1 \times m_k$ and represents the treatment indicator for the omitted cell in cluster k. Since a pair of cells is being omitted, $\sum_{k=1}^{K} m_k = 2$. Therefore, $\tilde{\mathbf{Z}}_o$ will be of dimension $(T+1) \times 2$.

Then $\tilde{\mathbf{Z}}_o\mathbf{D}^{-1}\tilde{\mathbf{Z}}_o^T$ can be written as

$$\begin{bmatrix} \sum_{k=1}^{K} \tilde{\mathbf{T}}_{m_k}(\mathbf{V}_{m_k} - \mathbf{C}^{\neq 0}_{m_k}\mathbf{V}^{-1}_{[m_k]}\mathbf{C}^{\neq 0T}_{m_k})^{-1}\tilde{\mathbf{T}}^T_{m_k} & \sum_{k=1}^{K} \tilde{\mathbf{T}}_{m_k}(\mathbf{V}_{m_k} - \mathbf{C}^{\neq 0}_{m_k}\mathbf{V}^{-1}_{[m_k]}\mathbf{C}^{\neq 0T}_{m_k})^{-1}\tilde{\mathbf{X}}^T_{m_k} \\ \sum_{k=1}^{K} \tilde{\mathbf{X}}_{m_k}(\mathbf{V}_{m_k} - \mathbf{C}^{\neq 0}_{m_k}\mathbf{V}^{-1}_{[m_k]}\mathbf{C}^{\neq 0T}_{m_k})^{-1}\tilde{\mathbf{T}}^T_{m_k} & \sum_{k=1}^{K} \tilde{\mathbf{X}}_{m_k}(\mathbf{V}_{m_k} - \mathbf{C}^{\neq 0}_{m_k}\mathbf{V}^{-1}_{[m_k]}\mathbf{C}^{\neq 0T}_{m_k})^{-1}\tilde{\mathbf{X}}^T_{m_k} \end{bmatrix}.$$

Then, recalling the expression for $\mathbf{Z}^T\mathbf{V}^{-1}\mathbf{Z}$ given in Section A, an expression for $\mathbf{Z}^T\mathbf{V}^{-1}\mathbf{Z} - \tilde{\mathbf{Z}}_o\mathbf{D}^{-1}\tilde{\mathbf{Z}}_o^T$ is available, and standard matrix algebra shows that the $(T+1) \times (T+1)$th entry of $(\mathbf{Z}^T\mathbf{V}^{-1}\mathbf{Z} - \tilde{\mathbf{Z}}_o\mathbf{D}^{-1}\tilde{\mathbf{Z}}_o^T)^{-1}$ is given by $\left(B_{22} - B_{21}B_{11}^{-1}B_{21}^T\right)^{-1}$, where

$$\begin{aligned} B_{22} &= \sum_{k=1}^{K} \mathbf{X}_k^T\mathbf{V}_k^{-1}\mathbf{X}_k - \sum_{k=1}^{K} \tilde{\mathbf{X}}_{m_k}(\mathbf{V}_{m_k} - \mathbf{C}^{\neq 0}_{m_k}\mathbf{V}^{-1}_{[m_k]}\mathbf{C}^{\neq 0T}_{m_k})^{-1}\tilde{\mathbf{X}}^T_{m_k} \\ B_{21} &= \left(\sum_{k=1}^{K} \mathbf{Z}_k^T\mathbf{V}_k^{-1}\mathbf{X}_k\right)^T - \sum_{k=1}^{K} \tilde{\mathbf{X}}_{m_k}(\mathbf{V}_{m_k} - \mathbf{C}^{\neq 0}_{m_k}\mathbf{V}^{-1}_{[m_k]}\mathbf{C}^{\neq 0}_{m_k})^{-1}\tilde{\mathbf{T}}^T_{m_k} \\ B_{11} &= \sum_{k=1}^{K} \mathbf{Z}_k^T\mathbf{V}_k^{-1}\mathbf{Z}_k - \sum_{k=1}^{K} \tilde{\mathbf{T}}_{m_k}(\mathbf{V}_{m_k} - \mathbf{C}^{\neq 0}_{m_k}\mathbf{V}^{-1}_{[m_k]}\mathbf{C}^{\neq 0T}_{m_k})^{-1}\tilde{\mathbf{T}}^T_{m_k}. \end{aligned}$$

Suppose that the pair of centrosymmetric cells belongs to clusters s and s'. These cells could either be in the same or in different clusters, and hence the expression $(\mathbf{V}_{m_k} - \mathbf{C}^{\neq 0}_{m_k}\mathbf{V}^{-1}_{[m_k]}\mathbf{C}^{\neq 0T}_{m_k})^{-1}$ can be written in one of the following two ways:

(1) Centrosymmetric pair belonging to different clusters

If the pair of centrosymmetric cells belongs to different clusters ($s \neq s'$), then $m_s = m_{s'} = 1$ and we can make the following simplifications: The terms $\mathbf{V}_{m_s} = \mathbf{V}_{m_{s'}} = a$, where a is a scalar and represents the variance of each of the omitted cluster-period cells, and $\tilde{\mathbf{X}}_{m_k}$ is also a scalar, which we will denote by $\tilde{x}_{m_k}$. The terms $\mathbf{C}^{\neq 0}_{m_s}\mathbf{V}^{-1}_{[m_s]}\mathbf{C}^{\neq 0T}_{m_s} = \mathbf{C}^{\neq 0}_{m_{s'}}\mathbf{V}^{-1}_{[m_{s'}]}\mathbf{C}^{\neq 0T}_{m_{s'}} = b$, where b is a scalar. So, $(\mathbf{V}_{m_s} - \mathbf{C}^{\neq 0}_{m_s}\mathbf{V}^{-1}_{[m_s]}\mathbf{C}^{\neq 0T}_{m_s})^{-1} = (a-b)^{-1} = f$, where f is a scalar. Furthermore, the summations in the second terms of each of B_{22}, B_{21}, and B_{11} can be simplified to sums of terms with cluster indices s and s' only.

$$B_{22} = \sum_{k=1}^{K} \mathbf{X}_k^T \mathbf{V}_k^{-1} \mathbf{X}_k - f\left(\tilde{\mathbf{x}}_{m_s}^2 + \tilde{\mathbf{x}}_{m_{s'}}^2\right)$$

$$= \sum_{k=1}^{K} \mathbf{X}_k^T \mathbf{V}_k^{-1} \mathbf{X}_k - g$$

$$B_{21} = \left(\sum_{k=1}^{K} \mathbf{Z}_k^T \mathbf{V}_k^{-1} \mathbf{X}_k\right)^T - f\left(\tilde{x}_{m_s} \tilde{\mathbf{T}}_{m_s}^T + \tilde{x}_{m_{s'}} \tilde{\mathbf{T}}_{m_{s'}}^T\right)$$

$$= \left(\sum_{k=1}^{K} \mathbf{Z}_k^T \mathbf{V}_k^{-1} \mathbf{X}_k\right)^T - \mathbf{h}$$

$$B_{11} = \sum_{k=1}^{K} \mathbf{Z}_k^T \mathbf{V}_k^{-1} \mathbf{Z}_k - f\left(\tilde{\mathbf{T}}_{m_s} \tilde{\mathbf{T}}_{m_s}^T + \tilde{\mathbf{T}}_{m_{s'}} \tilde{\mathbf{T}}_{m_{s'}}^T\right)$$

$$= \sum_{k=1}^{K} \mathbf{Z}_k^T \mathbf{V}_k^{-1} \mathbf{Z}_k - \mathbf{L}$$

where $g = f\left(\tilde{\mathbf{x}}_{m_s}^2 + \tilde{\mathbf{x}}_{m_{s'}}^2\right)$ is a scalar, $\mathbf{h} = f\left(\tilde{x}_{m_s} \tilde{\mathbf{T}}_{m_s}^T + \tilde{x}_{m_{s'}} \tilde{\mathbf{T}}_{m_{s'}}^T\right)$ is $1 \times T$ vector, and $\mathbf{L} = f\left(\tilde{\mathbf{T}}_{m_s} \tilde{\mathbf{T}}_{m_s}^T + \tilde{\mathbf{T}}_{m_{s'}} \tilde{\mathbf{T}}_{m_{s'}}^T\right)$ is a $T \times T$ matrix.

B_{22} can also be written as below by recalling the expression for $var(\hat{\theta})$ given in Section A:

$$\frac{1}{var(\hat{\theta})} = \sum_{k=1}^{K} \mathbf{X}_k^T \mathbf{V}_k^{-1} \mathbf{X}_k - \left(\sum_{k=1}^{K} \mathbf{Z}_k^T \mathbf{V}_k^{-1} \mathbf{X}_k\right)^T \left(\sum_{k=1}^{K} \mathbf{Z}_k^T \mathbf{V}_k^{-1} \mathbf{Z}_k\right)^{-1} \left(\sum_{k=1}^{K} \mathbf{Z}_k^T \mathbf{V}_k^{-1} \mathbf{X}_k\right)$$

$$\Rightarrow \frac{1}{var(\hat{\theta})} + \left(\sum_{k=1}^{K} \mathbf{Z}_k^T \mathbf{V}_k^{-1} \mathbf{X}_k\right)^T \left(\sum_{k=1}^{K} \mathbf{Z}_k^T \mathbf{V}_k^{-1} \mathbf{Z}_k\right)^{-1} \left(\sum_{k=1}^{K} \mathbf{Z}_k^T \mathbf{V}_k^{-1} \mathbf{X}_k\right) = \sum_{k=1}^{K} \mathbf{X}_k^T \mathbf{V}_k^{-1} \mathbf{X}_k$$

$$\Rightarrow \frac{1}{var(\hat{\theta})} + \left(\sum_{k=1}^{K} \mathbf{Z}_k^T \mathbf{V}_k^{-1} \mathbf{X}_k\right)^T \left(\sum_{k=1}^{K} \mathbf{Z}_k^T \mathbf{V}_k^{-1} \mathbf{Z}_k\right)^{-1} \left(\sum_{k=1}^{K} \mathbf{Z}_k^T \mathbf{V}_k^{-1} \mathbf{X}_k\right) - g = \sum_{k=1}^{K} \mathbf{X}_k^T \mathbf{V}_k^{-1} \mathbf{X}_k - g$$

Letting $var_{[o]}(\hat{\theta})$ be the variance of the treatment effect estimator for the included cells where the original design may be an incomplete design,

$$
\begin{aligned}
\frac{1}{var_{[o]}(\hat{\theta})} &= B_{22} - B_{21}B_{11}^{-1}B_{21}^{T} \\
\frac{1}{var_{[o]}(\hat{\theta})} &= \frac{1}{var(\hat{\theta})} + \left(\sum_{k=1}^{K}\mathbf{Z}_k^T\mathbf{V}_k^{-1}\mathbf{X}_k\right)^T \left(\sum_{k=1}^{K}\mathbf{Z}_k^T\mathbf{V}_k^{-1}\mathbf{Z}_k\right)^{-1} \left(\sum_{k=1}^{K}\mathbf{Z}_k^T\mathbf{V}_k^{-1}\mathbf{X}_k\right) - g \\
&\quad - \left(\left(\sum_{k=1}^{K}\mathbf{Z}_k^T\mathbf{V}_k^{-1}\mathbf{X}_k\right)^T - \mathbf{h}\right) \left(\sum_{k=1}^{K}\mathbf{Z}_k^T\mathbf{V}_k^{-1}\mathbf{Z}_k - \mathbf{L}\right)^{-1} \left(\sum_{k=1}^{K}\mathbf{Z}_k^T\mathbf{V}_k^{-1}\mathbf{X}_k - \mathbf{h}^T\right) \\
\frac{1}{var_{[o]}(\hat{\theta})} &= \frac{1}{var(\hat{\theta})} + C \\
\frac{1}{var_{[o]}(\hat{\theta})} &= \frac{1 + var(\hat{\theta}) \times C}{var(\hat{\theta})}
\end{aligned}
$$

Therefore, the information content of the centrosymmetric pair of cluster-period cells denoted by o is given by

$$
IC(o) = \frac{var_{[o]}(\hat{\theta})}{var(\hat{\theta})} = \frac{1}{1 + var(\hat{\theta}) \times C}
$$

where

$$
\begin{aligned}
C = &\left(\sum_{k=1}^{K}\mathbf{Z}_k^T\mathbf{V}_k^{-1}\mathbf{X}_k\right)^T \left(\sum_{k=1}^{K}\mathbf{Z}_k^T\mathbf{V}_k^{-1}\mathbf{Z}_k\right)^{-1} \left(\sum_{k=1}^{K}\mathbf{Z}_k^T\mathbf{V}_k^{-1}\mathbf{X}_k\right) - g \\
&- \left(\left(\sum_{k=1}^{K}\mathbf{Z}_k^T\mathbf{V}_k^{-1}\mathbf{X}_k\right)^T - \mathbf{h}\right) \left(\sum_{k=1}^{K}\mathbf{Z}_k^T\mathbf{V}_k^{-1}\mathbf{Z}_k - \mathbf{L}\right)^{-1} \left(\sum_{k=1}^{K}\mathbf{Z}_k^T\mathbf{V}_k^{-1}\mathbf{X}_k - \mathbf{h}^T\right).
\end{aligned}
$$

(2) Centrosymmetric pair belonging to the same cluster:

If the pair of centrosymmetric cells belongs to the same cluster ($s = s'$), then $m_s = 2$ and we can make the following simplifications: The terms $\mathbf{V}_{m_k}$ is a 2×2 matrix and represents the covariance of the omitted pair of cells from cluster k, and $\tilde{\mathbf{X}}_{m_k}$ is a 1×2-dimensional vector, which we will denote by $\tilde{\mathbf{x}}_{m_k}$. The term $\mathbf{C}_{m_k}^{\neq 0}\mathbf{V}_{[m_k]}^{-1}\mathbf{C}_{m_k}^{\neq 0T}$ is a 2×2 matrix. So, $\left(\mathbf{V}_{m_k} - \mathbf{C}_{m_k}^{\neq 0}\mathbf{V}_{[m_k]}^{-1}C_{m_k}^{\neq 0T}\right)^{-1} = \mathbf{F}$, where $\mathbf{F}$ is a 2×2 matrix.
Furthermore, the summations in the second terms of each of B_{22}, B_{21}, and B_{11} can be simplified to terms with cluster index s only.

$$
\begin{aligned}
B_{22} &= \sum_{k=1}^{K} \mathbf{X}_k^T \mathbf{V}_k^{-1} \mathbf{X}_k - \tilde{\mathbf{x}}_{m_s} \mathbf{F} \tilde{\mathbf{x}}_{m_s}^T \\
&= \sum_{k=1}^{K} \mathbf{X}_k^T \mathbf{V}_k^{-1} \mathbf{X}_k - n \\
B_{21} &= \left(\sum_{k=1}^{K} \mathbf{Z}_k^T \mathbf{V}_k^{-1} \mathbf{X}_k \right)^T - \tilde{\mathbf{x}}_{m_s} \mathbf{F} \tilde{\mathbf{T}}_{m_s}^T \\
&= \left(\sum_{k=1}^{K} \mathbf{Z}_k^T \mathbf{V}_k^{-1} \mathbf{X}_k \right)^T - \mathbf{p} \\
B_{11} &= \sum_{k=1}^{K} \mathbf{Z}_k^T \mathbf{V}_k^{-1} \mathbf{Z}_k - \tilde{\mathbf{T}}_{m_s} \mathbf{F} \tilde{\mathbf{T}}_{m_s}^T \\
&= \sum_{k=1}^{K} \mathbf{Z}_k^T \mathbf{V}_k^{-1} \mathbf{Z}_k - \mathbf{Q}
\end{aligned}
$$

$n = \left(\tilde{\mathbf{x}}_{m_s} \mathbf{F} \tilde{\mathbf{x}}_{m_s}^T \right)$ is a scalar , $\mathbf{p} = \left(\tilde{\mathbf{x}}_{m_k} \mathbf{F} \tilde{\mathbf{T}}_{m_k}^T \right)$ is a $1 \times T$ vector , and $\mathbf{Q} = \left(\tilde{\mathbf{T}}_{m_k} \mathbf{F} \tilde{\mathbf{T}}_{m_k}^T \right)$ is a $T \times T$ matrix.

B_{22} can also be written as below by recalling the expression for $var(\hat{\theta})$ given in Section A:

$$
\frac{1}{var(\hat{\theta})} = \sum_{k=1}^{K} \mathbf{X}_k^T \mathbf{V}_k^{-1} \mathbf{X}_k - \left(\sum_{k=1}^{K} \mathbf{Z}_k^T \mathbf{V}_k^{-1} \mathbf{X}_k \right)^T \left(\sum_{k=1}^{K} \mathbf{Z}_k^T \mathbf{V}_k^{-1} \mathbf{Z}_k \right)^{-1} \left(\sum_{k=1}^{K} \mathbf{Z}_k^T \mathbf{V}_k^{-1} \mathbf{X}_k \right)
$$

$$
\Rightarrow \frac{1}{var(\hat{\theta})} + \left(\sum_{k=1}^{K} \mathbf{Z}_k^T \mathbf{V}_k^{-1} \mathbf{X}_k \right)^T \left(\sum_{k=1}^{K} \mathbf{Z}_k^T \mathbf{V}_k^{-1} \mathbf{Z}_k \right)^{-1} \left(\sum_{k=1}^{K} \mathbf{Z}_k^T \mathbf{V}_k^{-1} \mathbf{X}_k \right) = \sum_{k=1}^{K} \mathbf{X}_k^T \mathbf{V}_k^{-1} \mathbf{X}_k
$$

$$
\begin{aligned}
\Rightarrow \frac{1}{var(\hat{\theta})} &+ \left(\sum_{k=1}^{K} \mathbf{Z}_k^T \mathbf{V}_k^{-1} \mathbf{X}_k \right)^T \left(\sum_{k=1}^{K} \mathbf{Z}_k^T \mathbf{V}_k^{-1} \mathbf{Z}_k \right)^{-1} \left(\sum_{k=1}^{K} \mathbf{Z}_k^T \mathbf{V}_k^{-1} \mathbf{X}_k \right) - n \\
&= \sum_{k=1}^{K} \mathbf{X}_k^T \mathbf{V}_k^{-1} \mathbf{X}_k - n
\end{aligned}
$$

Letting $var_{[o]}(\hat{\theta})$ be the variance of the treatment effect estimator for observed cells where the original design may be an incomplete design,

$$
\begin{aligned}
\frac{1}{var_{[o]}(\hat{\theta})} &= B_{22} - B_{21}B_{11}^{-1}B_{21}^{T} \\
\frac{1}{var_{[o]}(\hat{\theta})} &= \frac{1}{var(\hat{\theta})} + \left(\sum_{k=1}^{K} \mathbf{Z}_k^T \mathbf{V}_k^{-1} \mathbf{X}_k\right)^T \left(\sum_{k=1}^{K} \mathbf{Z}_k^T \mathbf{V}_k^{-1} \mathbf{Z}_k\right)^{-1} \left(\sum_{k=1}^{K} \mathbf{Z}_k^T \mathbf{V}_k^{-1} \mathbf{X}_k\right) - n \\
&\quad - \left(\left(\sum_{k=1}^{K} \mathbf{Z}_k^T \mathbf{V}_k^{-1} \mathbf{X}_k\right)^T - \mathbf{p}\right) \left(\sum_{k=1}^{K} \mathbf{Z}_k^T \mathbf{V}_k^{-1} \mathbf{Z}_k - \mathbf{Q}\right)^{-1} \left(\sum_{k=1}^{K} \mathbf{Z}_k^T \mathbf{V}_k^{-1} \mathbf{X}_k - \mathbf{p}^T\right) \\
\frac{1}{var_{[o]}(\hat{\theta})} &= \frac{1}{var(\hat{\theta})} + C^* \\
\frac{1}{var_{[o]}(\hat{\theta})} &= \frac{1 + var(\hat{\theta}) \times C^*}{var(\hat{\theta})}
\end{aligned}
$$

Therefore, the information content of the centrosymmetric pair of cluster-period cells denoted by o is given by

$$
IC(o) = \frac{var_{[o]}(\hat{\theta})}{var(\hat{\theta})} = \frac{1}{1 + var(\hat{\theta}) \times C^*}
$$

where

$$
\begin{aligned}
C^* = &\left(\sum_{k=1}^{K} \mathbf{Z}_k^T \mathbf{V}_k^{-1} \mathbf{X}_k\right)^T \left(\sum_{k=1}^{K} \mathbf{Z}_k^T \mathbf{V}_k^{-1} \mathbf{Z}_k\right)^{-1} \left(\sum_{k=1}^{K} \mathbf{Z}_k^T \mathbf{V}_k^{-1} \mathbf{X}_k\right) - n \\
&- \left(\left(\sum_{k=1}^{K} \mathbf{Z}_k^T \mathbf{V}_k^{-1} \mathbf{X}_k\right)^T - \mathbf{p}\right) \left(\sum_{k=1}^{K} \mathbf{Z}_k^T \mathbf{V}_k^{-1} \mathbf{Z}_k - \mathbf{Q}\right)^{-1} \left(\sum_{k=1}^{K} \mathbf{Z}_k^T \mathbf{V}_k^{-1} \mathbf{X}_k - \mathbf{p}^T\right)
\end{aligned}
$$

C Plots of the information content for the larger designs

More subjects (50) and larger ICC (0.05)

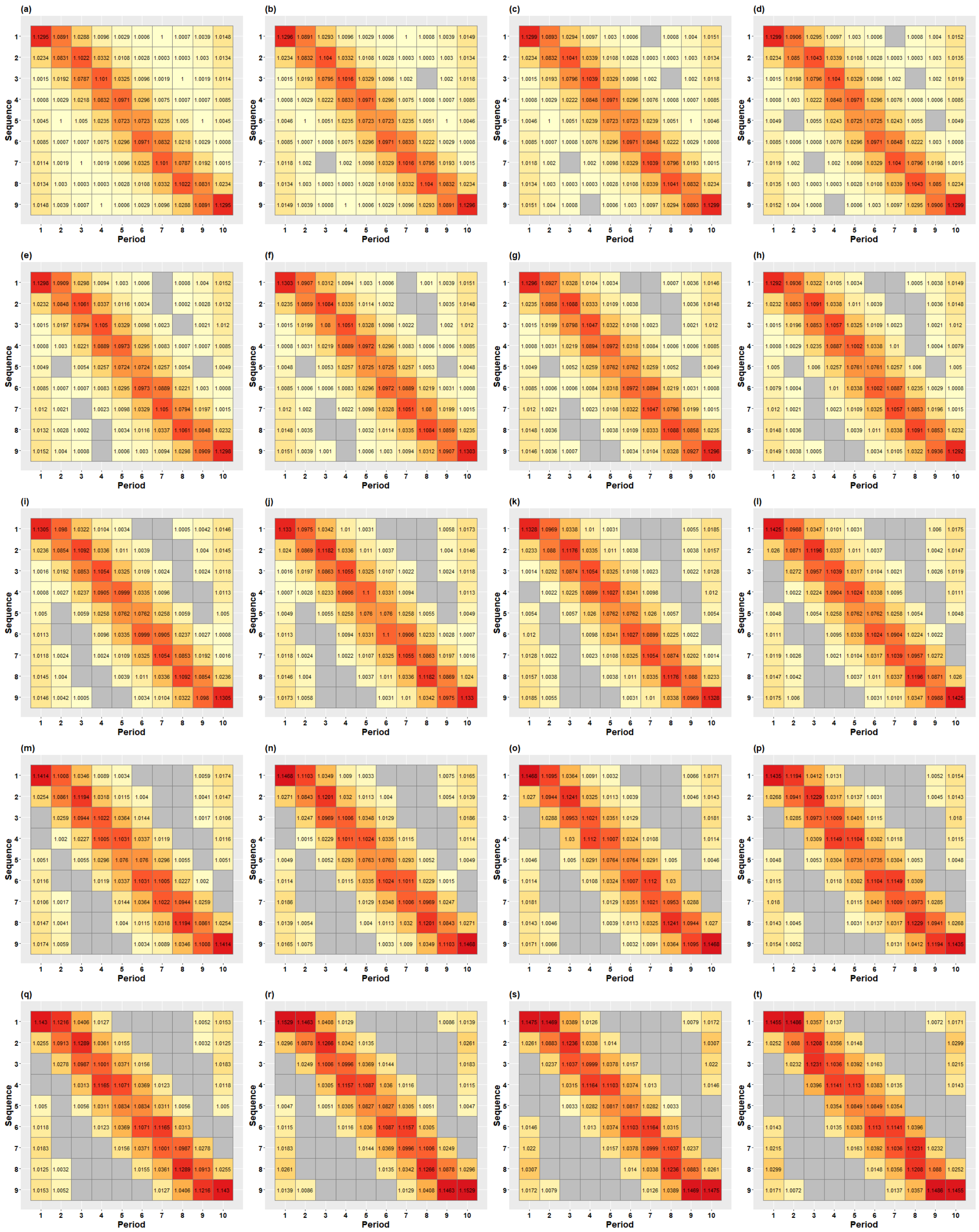

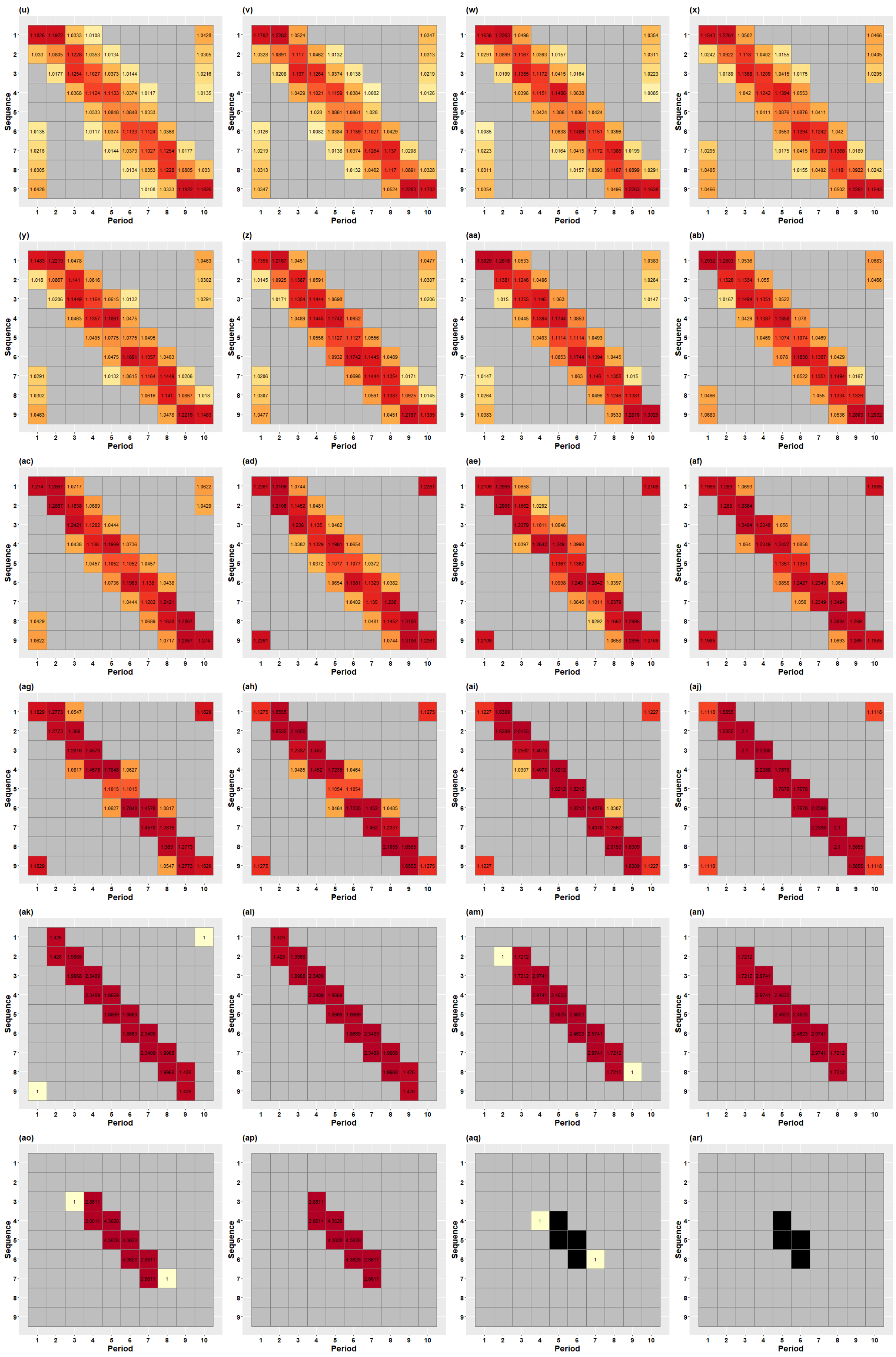

Figure C1: Information content of the cells in progressively reduced designs, with 50 subjects per cell, assuming a discrete-time decay model with intracluster correlation of 0.05, and cluster autocorrelation of 0.95. The black color indicates that no information content can be calculated for the centrosymmetric cell pair.

Small number of subjects (10) and small ICC (0.01)

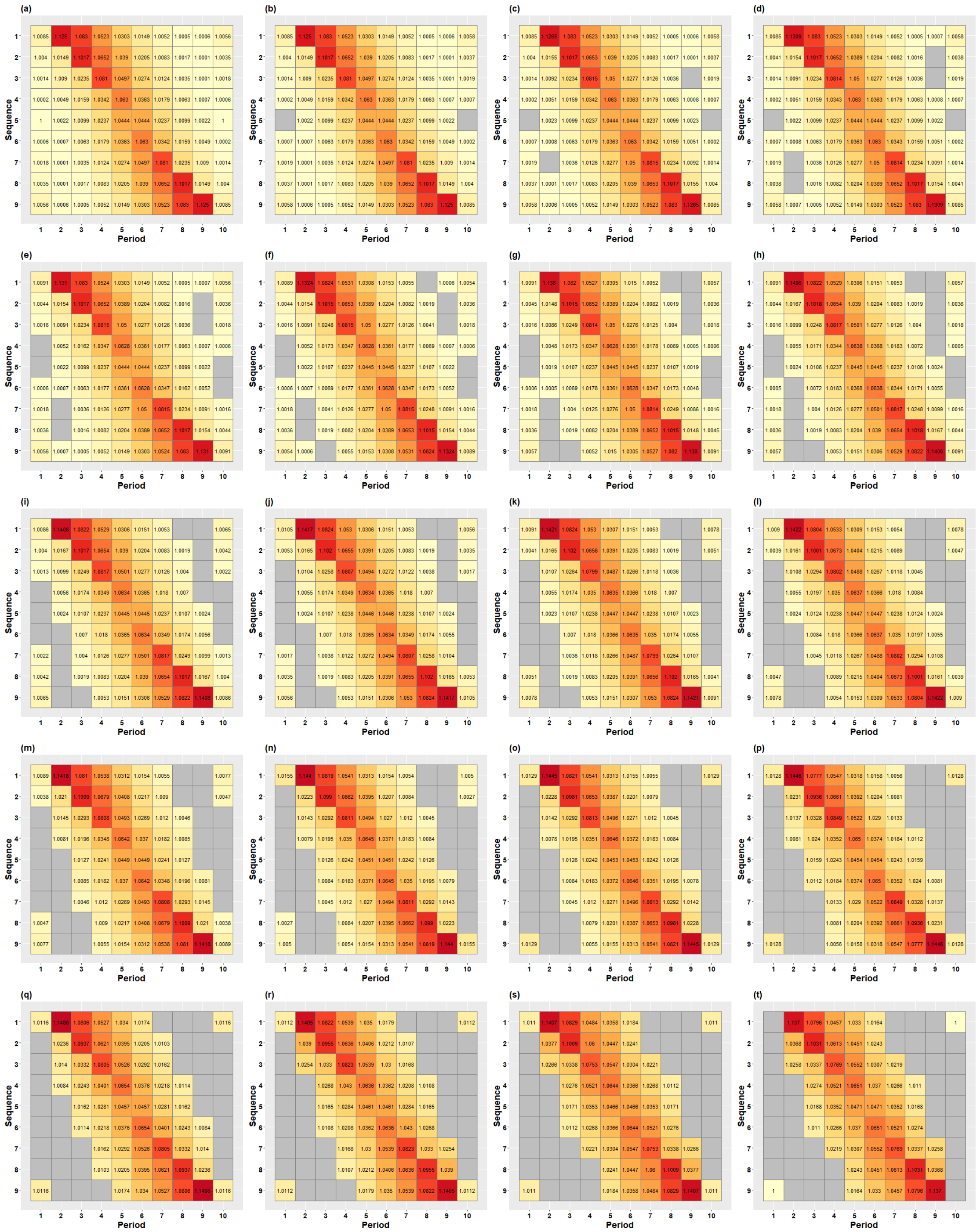

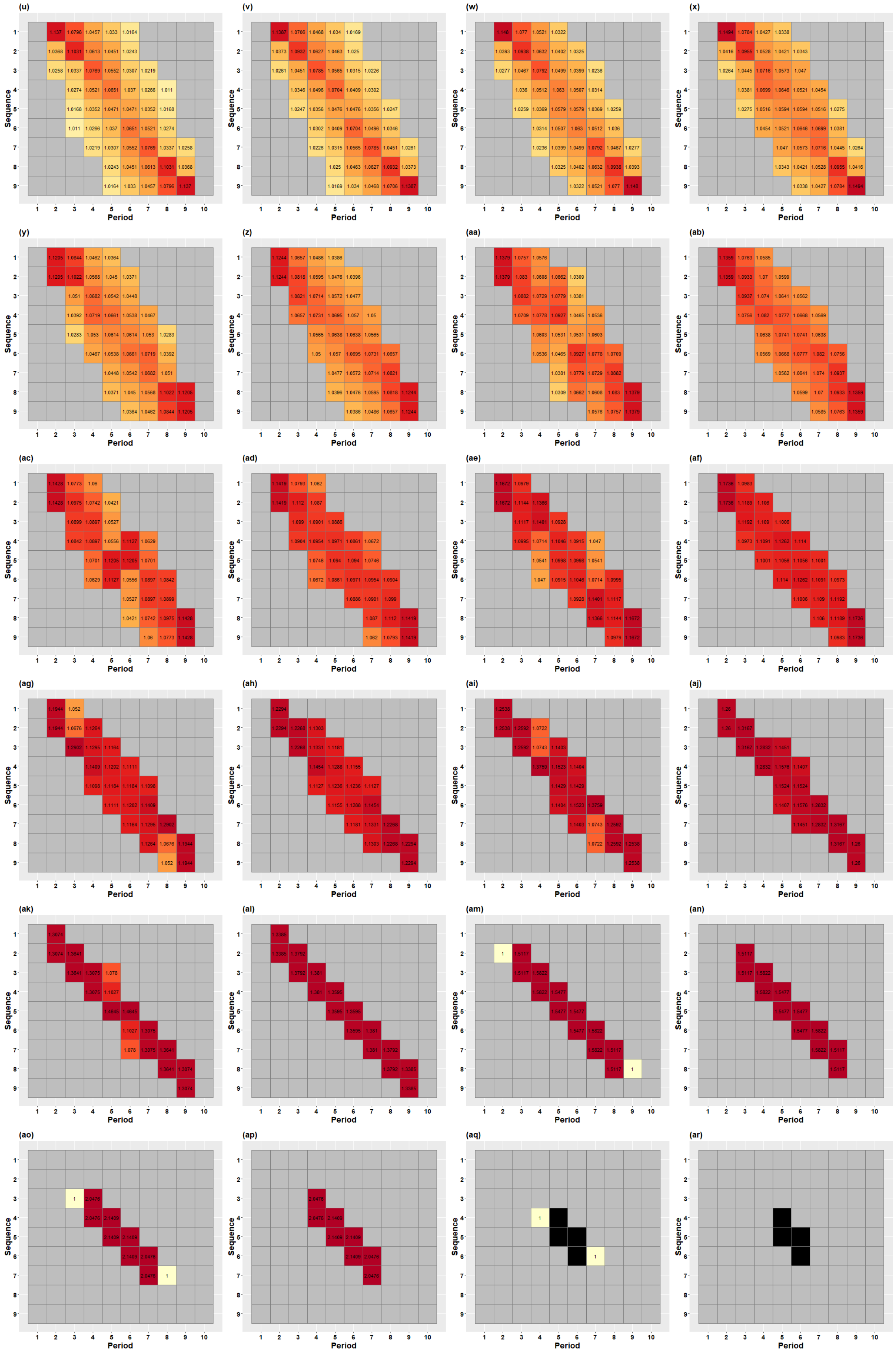

Figure C2: Information content of the cells in progressively reduced designs, with 10 subjects per cell, assuming a discrete-time decay model with intracluster correlation of 0.01, and cluster autocorrelation of 0.95. The black color indicates that no information content can be calculated for the centrosymmetric cell pair.

References

[1] Kasza J, Hemming K, Hooper R, Matthews J, Forbes AB. Impact of non-uniform correlation structure on sample size and power in multiple-period cluster randomised trials. Stat Methods Med Res. 2019;28(3):703–716.
